# Unravelling the transcriptomic dynamics of *Hyphopichia pseudoburtonii* in co-culture with *Botrytis cinerea*

**DOI:** 10.1371/journal.pone.0316713

**Published:** 2025-01-14

**Authors:** Evelyn Maluleke, Neil Paul Jolly, Hugh-George Patterton, Mathabatha Evodia Setati

**Affiliations:** 1 South African Grape and Wine Research Institute, Stellenbosch University, Stellenbosch, South Africa; 2 Post Harvest and Agro-Processing Technologies, ARC Infruitec-Nietvoorbij (The Fruit, Vine and Wine Institute of the Agricultural Research Council), Stellenbosch, South Africa; 3 Centre for Bioinformatics and Computational Biology, Stellenbosch University, Stellenbosch, South Africa; University of California Riverside, UNITED STATES OF AMERICA

## Abstract

*Hyphopichia pseudoburtonii*, is emerging as a potential biocontrol agent against various phytopathogens. These traits have been attributed to the production of various antifungal compounds in the presence of target pathogens. However, the broad molecular mechanisms involved in the antifungal activity are not yet understood. This study employed RNA sequencing to assess the temporal changes in *H*. *pseudoburtonii* Y963 gene expression patterns when co-cultivated with *Botrytis cinerea*. Genes differentially expressed in *H*. *pseudoburtonii* in co-culture with *B*. *cinerea*, compared to the monoculture were evaluated after 24, 48, and 120 h of growth. Up-regulation of genes encoding major core histones (H2A, H3, H4) and ribosomes in the first 24 h suggested an abundance of cells in the S phase of the cell cycle. At 48 h, the genes up-regulated highlight mitotic cell cycle activity and induction of filamentous growth, while in later stages, up-regulation of genes encoding high affinity transporters of sugars, copper and iron, as well as those involved in the retention and utilization of siderophore-iron was evident. Altogether, the data allude to competition for space and nutrients as key mechanisms activated in *H*. *pseudoburtonii* in the presence of *B*. *cinerea*. This research offers new insights into *H*. *pseudoburtonii* transcriptomic response to *B*. *cinerea* and illuminates the adaptive strategies and molecular mechanisms behind its antifungal activity.

## Introduction

*Hyphopichia pseudoburtonii* is a non-conventional mycelial yeast that forms pseudo-hyphae and septate hyphae in response to varying growth conditions [1, 2]. This yeast is widely distributed in nature and has been found in different environments, including rumen contents [3], bread [4], sourdough [5], grapes and grape must [6–9], Nuruk [2, 10, 11], mites [12], fish intestinal tract [13], bird feathers [14], and potato peel waste [15].

Some strains of *H*. *pseudoburtonii* can metabolize different types of sugars including sucrose, d-glucose, d-galactose, maltose and trehalose [1]. However, the fermentative capacity of this species for d-glucose has been reported to be lower compared to *Saccharomyces cerevisiae* strains [16], and it can tolerate up to 7% ethanol [15, 16]. *H*. *pseudoburtonii* has been found to possess antifungal properties and display broad-spectrum inhibition against *B*. *cinerea* [17, 18], as well as other pathogens such as *Penicillium expansum* [16], *Alternaria alternata* and *Aspergillus niger* [18]. Certain strains of *H*. *pseudoburtonii* have the ability to release cell wall lytic enzymes when exposed to *Botrytis cinerea* [18] and β-1,4-cellulases when cultivated on cellulose [6], while others form biofilm on fruit wound sites [17]. Previous research demonstrated that these characteristics are important in yeasts for biocontrol activity against plant pathogens [19, 20]. Recent studies also suggest that certain strains of *H*. *pseudoburtonii* may have the ability to promote plant growth [8]. For instance, a strain isolated from *Vitis vinifera* grapes was found to significantly enhance the development of *Nicotiana benthamiana* seedlings, both above and below ground, resulting in an overall increase in dry weight [8].

The biocontrol potential of *H*. *pseudoburtonii* has mainly been investigated using phenotypic traits, while the molecular mechanisms underpinning its antifungal activity and interactions with target phytopathogens have not been investigated. Various experimental approaches have been employed to investigate the molecular mechanisms involved in both two-way (pathogen/antagonist) interactions [21, 22] or three-way (pathogen/antagonist/host) interactions [23–25]. Omics techniques, such as proteome and transcriptome analyses, have provided insights into the complex dynamics of biological control systems [21, 26–28]. These studies have expanded our knowledge of biocontrol mechanisms involving yeast and pathogens by revealing genes and pathways associated with biocontrol mechanisms such as genes involved in hydrolases, cell wall structure and integrity, competition for trace elements (zinc and copper) and carbohydrates (glucose). Instead of solely identifying specific molecules or interactions, these investigations have also explored the complex molecular dynamics and broader cellular responses involved in yeast-pathogen interactions. Obtaining a thorough understanding of how biological control agents work is crucial for maximizing their potential, ensuring safety, and promoting successful commercialization [29–31]. In our previous work, we highlighted the antifungal activity of a grape must derived *H*. *pseudoburtonii* Y963 strain against various phytopathogens including different strains of *B*. *cinerea* [18]. Our findings, revealed reduction on glucan and chitin levels in the cell wall of *B*. *cinerea* hyphae when challenged with *H*. *pseudoburtonii*, seemingly associated with the production of cell wall degrading enzymes, cyclic peptides damage cell wall constituents as well as volatile organic compounds that inhibit spore germination. Here we used transcriptomic and proteomic analyses to profile the genetic mechanisms underlying the interactions between *H*. *pseudoburtonii* and *B*. *cinerea* in liquid co-cultures. The aim was to identify potential dominant molecular signatures that could explain the antagonistic activity of *H*. *pseudoburtonii* against *B*. *cinerea*. We hypothesized based on our previous findings [18] that genes and proteins related to antifungal compounds production, inhibition of cell wall biogenesis and integrity, or inhibition of spore germination would be up-regulated in *H*. *pseudoburtonii* in the presence of *B*. *cinerea*.

## Materials and methods

### Microbial strains and culture media

*H*. *pseudoburtonii* Y963 and *B*. *cinerea* FF1, were obtained from the culture collection of the South African Grape and Wine Research Institute (SAGWRI) at Stellenbosch University. *H*. *pseudoburtonii* Y963 was regularly cultivated and maintained on Wallerstein Nutrient (WLN) agar (Merck Millipore, South Africa). For extended storage periods, the strains were preserved at -80°C in cryogenic tubes containing a 25% (v/v) glycerol solution. *B*. *cinerea* FF1 was revived and grown on Malt Extract agar (MEA; Merck Millipore, South Africa) containing 2% (w/v) bacteriological agar.

### Co-culture preparation

*B*. *cinerea* FF1 was inoculated on a Malt extract agar (MEA; Merck Millipore, South Africa) containing 20 g/L bacteriological agar, 30 g/L malt extract and 5 g/L mycological peptone and incubated for 7 days in the dark at 25°C, followed by 3 days under continuous light to enhance sporulation. A sterile bent glass Pasteur pipette and sterile distilled water containing 0.001% Tween-80 (Merck Millipore, South Africa) were used to harvest the mycelium-conidia from the agar into microcentrifuge tubes. The suspensions were homogenized by vortexing to release conidia from the mycelia and filtered through sterile glass-wool filter tips. Spore suspensions (∼10^6^ spores/mL) were prepared following spore counting on a Haemocytometer (Hausser Scientific, England). The inoculum from *H*. *pseudoburtonii* was prepared from an overnight culture grown in 5 mL YPD (Merck Millipore, South Africa) broth (10 g/L yeast extract, 20 g/L peptone and 20 g/L glucose). Fresh yeast culture was collected by centrifugation at 10,625 x *g* for 5 min and washed twice with sterile 0.9% (w/v) NaCl solution. The cell concentrations were adjusted to OD_600_ 0.1 (≈ 10^6^ CFU/mL) using 0.9% (w/v) NaCl.

*H*. *pseudoburtonii* Y963 was co-inoculated at a ratio of 1:1 with *B*. *cinerea* FF1 in 2 mL malt extract broth in 24-well plates at a starting concentration of 5 x 10^3^ cells/mL. Monocultures of *H*. *pseudoburtonii* and *B*. *cinerea* served as controls. The cultures incubated at 25°C, and samples (the full 2 mL) were withdrawn at 0, 24, 48 and 120 h. In the co-culture samples (*H*. *pseudoburtonii + B*. *cinerea*), no attempt was made to filter out *B*. *cinerea* since *H*. *pseudoburtonii* has a tendency to form pseudohyphae and true hyphae, thus making it difficult to separate the two organisms. Samples were subsequently centrifuged for 10 min at 15000 x *g* and the cells were used for RNA extraction. To compare the yeast biomass in the mono- and co-culture, samples taken at 0, 24, 48, and 120 h were plated on Wallerstein Nutrient Agar (WLN; Merck Millipore, South Africa) supplemented with 300 mg/L biphenyl and incubated at 25°C. The amount of viable culturable yeast cell were represented as colony forming units (CFU/mL).

### Total RNA extraction, cDNA synthesis and sequencing

#### RNA-extraction

**Fig 1:**
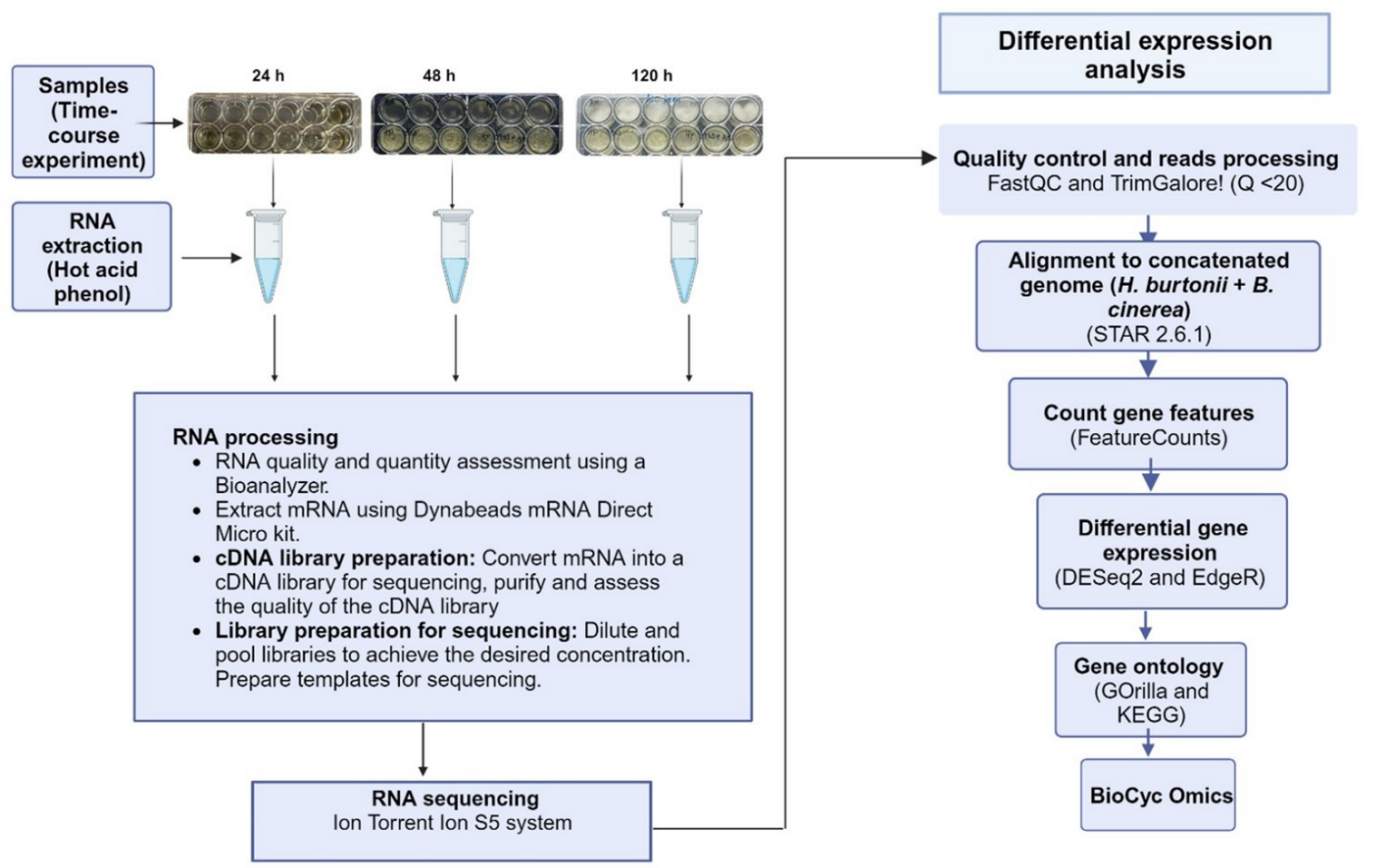
The experimental workflow for RNA-seq analysis of *H*. *pseudoburtonii* co-cultured with *B*. *cinerea* showing the sampling plan, RNA extraction and processing, as well as the data analysis steps.

Three biological replicates (Fig 1) were used for the extraction of high-quality total RNA using a modified version of the hot acidic phenol method originally described by Collart and Oliviero [32]. A modification was made for *H*. *pseudoburtonii* monoculture (control sample) and co-culture cells, in that 100 μL of acid-washed beads were added and the cells were vortexed for 45 s. Additionally, for all the samples, RNA was precipitated overnight at -20°C. The RNA was collected by centrifugation at 15000 x *g* for 10 min at 4°C and washed with 1 mL 75% (v/v) ethanol (Merck Millipore, South Africa). The RNA was air-dried in a laminar flow cabinet and then resuspended in 50 μL diethyl pyrocarbonate (Merck Millipore, South Africa)-treated dH_2_O.

#### RNA-sequencing

Total RNA from three biological replicates for each treatment (*H*. *pseudoburtonii* monoculture and *H*. *pseudoburtonii* + *B*. *cinerea*) were evaluated for integrity and quantity on the Bioanalyzer 2100 (Agilent Technologies, Waldbronn, Germany) utilizing the RNA 6000 Nano Chip and corresponding reagents. Subsequently, mRNA was isolated from 800 ng total RNA using the Dynabeads^™^ mRNA DIRECT^™^ Micro Purification Kit (Thermo Fisher Scientific, South Africa). Following this, the captured mRNA was attached to Dynabeads oligo (dT) 25, subjected to washing, and eventually eluted in 15 mL of nuclease-free water. The Ion Total Transcriptome Sequencing Kit v2 (Thermo Fisher Scientific, South Africa) was then used to synthesize cDNA for sequencing on the Ion Torrent Ion S5 system. The cDNA library was purified and assessed on the Agilent Bioanalyzer 2100 for yield and fragment size distribution using a high-sensitivity DNA chip and kit (Agilent Technologies, California, United States). The libraries were then diluted to achieve a target concentration of 80 pM and combined in equimolar quantities for template preparation using the Ion 540 Chef kit (Thermo Fisher Scientific, South Africa). Subsequently, enriched ion sphere particles were loaded onto an Ion 540 chip (Thermo Fisher Scientific, South Africa) and subjected to massively parallel sequencing on the Ion Torrent GeneStudio S5 Prime system, employing sequencing solution reagents as outlined in the manufacturer’s protocol. Standard analysis parameters in Torrent Suite version 5.12.2 software were applied for flow space calibration and basecaller analysis.

#### RNA-seq processing

Fig 1 depicts the pipeline followed from sample preparation to RNA-seq data analysis. Sequencing reads were checked for quality and adaptor contamination before and after trimming with FastQC 0.11.9 [33]. Low quality reads (Q <20) polyA reads, and ambiguous reads (containing N) were trimmed from the raw sequencing reads with Trim Galore 0.6.5 [33]. Additionally, reads shorter than 20 nt were removed with FastQC, and adapters on the remaining reads were trimmed with Cutadapt 1.7.1 [34].

#### RNA-seq analysis

The processed and trimmed reads were mapped against a concatenated genome representing two species: *Hyphopichia burtonii* (GCA_001661395.1) and *B*. *cinerea* (GCF_000143535.2). The genome assembly for *H*. *pseudoburtonii* is available on GenBank, however no reference annotation is available. The genomes of *H*. *burtonii* and *H*. *pseudoburtonii* have 85.17% nucleotide identity and significant divergence in synteny. In terms of functional comparison, both *Hyphopichia* genomes possess extended gene families of amino acid permeases and ATP-binding cassette (ABC) transporters, which are involved in nutrient uptake and transport. The individual reference genomes were obtained from NCBI (https://www.ncbi.nlm.nih.gov/) and concatenated using the cat command in STAR 2.6.1 [35]. Trimmed reads were mapped to the concatenated genome using STAR 2.6.1 [35]. During the alignment process, the default parameters of STAR were utilized, allowing for a maximum of 10 mismatches (—outFilterMismatchNmax 10) [35]. Subsequently, low-quality reads (≤ 20), as well as non-uniquely mapped reads were filtered using samtools 1.5 [36]. The resulting bam files were accompanied by a feature file, consisting of *H*. *burtonii* and *B*. *cinerea* reference genomes in gff format, and used to determine the number of reads mapping to a specific gene, based on the reference genome and gff annotation. The RNA-Seq data is available at NCBI under the GEO accession GSE267877 (https://www.ncbi.nlm.nih.gov/geo/query/acc.cgi?acc=GSE267877). The percentage reads mapped to both sides of the concatenated genome increased over time: 64.89% at 24 h, 87.83% at 48 h, and 88.48% at 120 h. The GeneCounts function from STAR was used to obtain read counts. Unknown genes in *H*. *pseudoburtonii* were identified through homology comparison with the *S*. *cerevisiae* S288C genome using Geneious Prime 2023.1 (https://www.geneious.com). Most genes associated with the translation of leader peptide codons in *H*. *pseudoburtonii* did not match any homologs identified in *S*. *cerevisiae* S288C.

#### Differential gene expression and functional analysis

To quantify the transcription levels and identify differentially expressed genes between the monoculture and co-cultures, a web server tool called Integrative Differential Expression Analysis for Multiple Experiments (IDEAMEX) [37] was used. The RNA-Seq raw count table in text format, generated using Subread-featureCounts, was uploaded onto the server. Differentially expressed genes were generated using the Bioconductor packages DESeq2 [38] and edgeR [39]. Genes were filtered by CPM expression using edgeR default settings and DESeq2 [38, 39]. Principal component analysis (PCA) was performed on the log-transformed count table. Pairwise comparisons were performed for monocultures and co-cultures. Fold changes (FC) were calculated, and *p*-values were adjusted for multiple comparisons (method “Benjamini-Hochberg”). Genes with an adjusted p-value < 0.05 and a log2FoldChange ratio ≥ 1 were defined as differentially expressed genes (DEGs). Gene expression patterns associated with each time point were analyzed on the BioCyc Omics Dashboard (https://biocyc.org/web-services.shtml) using *S*. *cerevisiae* S288C database. Through a systematic comparison of expression patterns across different subsystems and enzymes, meaningful trends were discerned, contributing to a comprehensive understanding of the biological dynamics under investigation. Gene Ontology (GO) analysis was performed on the differentially expressed genes using the Gene Ontology enrichment analysis and visualization (GOrilla) tool [40]. Enrichment analysis was based on the identified orthologs from *S*. *cerevisiae* S288C. As a result, genes unique to *H*. *pseudoburtonii*, including 133 genes, 15 genes, and 74 genes from the 24 h, 48 h, and 120 h DEGs categorized as hypothetical proteins and were excluded from this analysis. This exclusion may overlook species-specific biological processes and pathways. The GO terms obtained through GOrilla were summarised and visualized using REVIGO [41] to reduce redundancy and aid in interpretation. Visualization of the obtained DEGs was done using VENN diagrams generated on https://bioinformatics.psb.ugent.be/webtools/Venn platform.

### Proteomic analysis of *H*. *pseudoburtonii* co-cultured with *B*. *cinerea*

#### Protein extraction

To complement the RNAseq data, culture supernatants were collected from the 48-hour *H*. *pseudoburtonii* monocultures as well as the co-culture with *B*. *cinerea* to study the proteins accumulated in the extracellular environment. The 48 h cultures were centrifuged at 10,625 g for 10 minutes. The resulting supernatants were then filtered through a 0.22 μm syringe filter (Axiofilter, Axiology labs, Three Rivers, Vereeniging) and stored at -20°C for subsequent protein analysis.

#### In-solution digest

All reagents utilized were of analytical grade or of an equivalent quality. The filtered supernatants from *H*. *pseudoburtonii* Y963 as well as the co-cultures with *B*. *cinerea* IWBT FF1, underwent in-solution tryptic digestion by adding 1 μL of 50 mM triscarboxyethyl phosphine (TCEP; Fluka) prepared in 50 mM triethylammonium bicarbonate (TEAB). The samples were incubated for 1 h at 60°C and followed by cooling to room temperature. Subsequently, for alkylation, 1 μL of 100 mM S-Methyl methanethiosulphonate (MMTS) prepared in 2-propanol was added and incubated for 15 min at room temperature. The final volume was adjusted to 95 μL using 50 mM TEAB. For trypsination, trypsin (Promega, Madison, WI) was added to the sample at a 1:20 ratio (i.e., 5 μg/100 μg). The tubes were vortexed while securely sealed with parafilm to prevent evaporation and then incubated at 37°C for 18 h. For final extraction, samples were dried under vacuum in a SpeedVac and then resuspended in 30 μL of 2% acetonitrile/water; 0.1% formic acid. Residual digest reagents were eliminated using an in-house manufactured C18 stage tip (Empore Octadecyl C18 extraction discs; Supelco). The samples were loaded onto the stage tip after activation and equilibration of the C18 membrane with 3 μL methanol (Sigma) and 30 μL 2% acetonitrile:water; 0.05% trifluoroacetic acid (TFA), respectively. The bound samples were washed with 30 μL 2% acetonitrile:water; 0.1% TFA before elution with 30 μL 50% acetonitrile:water 0.05% TFA. The eluate was air-dried and subsequently dissolved into 2% acetonitrile:water; 0.1% formic acid for LC-MS/MS analysis.

#### Liquid chromatography

Samples were analysed using a Dionex Ultimate 3000 RSLC nano LC (Thermo Scientific; Massachusetts, USA) system coupled to a Thermo Scientific Fusion Orbitrap Mass Spectrometer equipped with a Nanospray Flex ionization source. Samples were loaded (mobile phase A: 2% acetonitrile with 0.1% formic acid) onto a C18 trapping column (Thermo Scientific; 5 mm × 300 μm, 5 μm; pore size 100 Å) and a Luna C18 analytical column (Phenomenex; 350 mm × 75 μm, 3.6 μm). Samples were loaded onto the trap column at a loading-pump flow rate of 15 μL/min from a temperature controlled autosampler set at 7°C for 5 min before eluting onto the analytical column. Peptide separation was performed at 40°C, at a flowrate of 250 nL/min and the gradient generated as follows: 5.0%-35%B over 60 min and 35%-50%B from 60–75 min and the outflow delivered to the mass spectrometer electro-spray interface (ESI).

#### Mass spectrometry

Mass spectrometry was performed using a Thermo Scientific Fusion mass spectrometer equipped with a Nanospray Flex ESI ionization source. The samples were introduced through a stainless-steel emitter. Data were collected in positive mode with spray voltage set to 1.8 kV and ion transfer capillary set to 280°C. Spectra were internally calibrated using polysiloxane ions at m/z = 445.12003 and 371.10024. MS1 scans were performed using the orbitrap detector set at 120 000 resolution over the scan range 375–1500 with automatic gain control (AGC) target at 5.0e4. Data was acquired in profile mode. MS2 acquisitions were performed using monoisotopic precursor selection for ion with charges state between states between 2+ and 7+. Undetermined charge states and charge states > 24 were excluded. Dynamic exclusion was conducted with mass error tolerance of ± 10 ppm with isotopes excluded after 1 time. Precursor ions were excluded from fragmentation once detected for a period of 60 s. Precursor ions were selected for fragmentation in HCD mode using the quadrupole mass analyser with HCD energy set to 30%. Fragment ions (MS2) were detected in the orbitrap mass analyser set to 30 000 resolution. The AGC target was set to 5.0e4 and the maximum injection time to 80 milliseconds, whereby ions were allowed to accumulate for a maximum of 80 milliseconds before being transferred to the mass analyser for measurements. The data was acquired in centroid mode, whereby the instrument recorded the mass and intensity of individual ions as distinct data points. Centroid mode captured the precise location of each ion peak in the acquired spectra, allowing for accurate quantification and subsequent analysis.

#### Data processing and analysis

The raw spectrum files generated were imported into proteome discoverer version 1.4.1.14 (Thermo Scientific, USA) and spectra were filtered using a minimum and maximum precursor mass of 350 and 5000 Da respectively with a threshold peak count of 15. Precursor and fragment masses were set to 20 ppm and 0.02 Da, respectively, with a maximum of 2 missed tryptic cleavages allowed. Peak lists were searched against a UniProtKB 2023_02 (https://www.uniprot.org/) database concatenated with the common repository of adventitious proteins (cRAP) using a sequential alternating SequestHT/MSAmanda [42, 43] search engine schema that included added amino acid modifications for each new cycle. Files from Proteome Discoverer software (.msf) were imported into Scaffold 5.2.0 Proteome Software (https://www.proteomesoftware.com/products/scaffold- 5) for data validation using X!Tandem. Final spectrum and peptide matching validation was conducted using Peptide Prophet and Protein Prophet Algorithms with the false discovery rate (FDR) for protein and peptides set to 1% and 0.1%, respectively. Proteins were identified with a minimum of 95% probability and ≥ 3 peptides. Relative quantitation was performed using the reporter ions quantifier built into Scaffold with yeast monoculture (controls) replicate samples set as the reference group. Proteins with a global false discovery rate (FDR) based on the Scaffold Local FDR algorithm, below 1.0% were considered as trusted proteins. The fold change (FC) of the trusted proteins and the significant difference p-value of the comparison group were calculated using the two-tailed t-test. Unique proteins detected only in the co-culture and those with a fold change ≥ 1.2 and p < 0.05 in the co-culture compared to the monoculture were considered to be differentially abundant proteins (DAPs).

#### Bioinformatic analysis of proteins

Differentially abundant and unique proteins were selected from the original dataset of the protein search results, and further analysed. STRING Version 11.5: functional protein association networks (https://string-db.org/) was used for functional annotation of the proteins and to identify potential interactions [44]. The main metabolic pathways associated with the identified proteins were identified using string-db.org which utilizes the information present in the KEGG database (http://www.genome.jp/kegg/) and integrates it with protein-protein interactions to identify proteins associated with specific pathways. This integration of data sources and computational methods enhances the accuracy and coverage of the generated pathways.

### Effect of iron on antifungal activity

Transcriptomic data revealed an up-regulation of several genes encoding proteins involved in iron sequestration. In order to investigate if *H*. *pseudoburtonii* indeed sequestered iron we screened for the influence of iron on the antagonistic activity. Firstly, potato dextrose agar (pH 4.5 and 5.5) plates supplemented with 5 and 20 μg/mL of FeCl_3_ were prepared and inoculated with *B*. *cinerea* spore suspension at 10^5^ spores/mL and 10 μL of yeast cell suspension at 10^6^ CFU/mL [7, 45]. Secondly, *H*. *pseudoburtonii* and *B*. *cinerea* were cultured in 24-well plates in malt extract broth in mono- and mixed-cultures as conducted for the transcriptomic analysis. Cultures were incubated at 25°C and samples were withdrawn at 0, 24, 48 and 120 h. The supernatants were analysed for the production of hydroxamate-type siderophores as described by Calvente et al. [46]. Briefly, 0.25 μL of the supernatants was mixed with 1.25 mL of FeCl_3_-0.1 M HClO_4_ (pH 2) in a cuvette followed by absorbance measurement at 480 nm.

## Results

### Growth dynamics

In the current study, an RNA sequencing approach was used to investigate the transcriptomic response of *H*. *pseudoburtonii* to the presence and possible growth of *B*. *cinerea* in liquid co-culture conditions. Monocultures were used as controls. Microscopic evaluation of the co-cultures compared to the monocultures of *H*. *pseudoburtonii* and *B*. *cinerea* revealed formation of yeast cells and hyphae in the co-culture (S1A Fig). Throughout the incubation period, substantial mycelium growth was consistently observed in the *B*. *cinerea* monoculture, while *H*. *pseudoburtonii* displayed budding cells at 24 h, and transitioning to pseudohyphae at 48 h. In contrast, when co-cultured together, *H*. *pseudoburtonii* cells were more abundant with budding cells at 24 h and a pronounced hyphal presence by 48 and 120 h. *H*. *pseudoburtonii* displayed similar growth dynamics and generated the same amount of biomass in both mono- and co-cultures (S1B Fig).

### Global analysis of the transcriptome

**Fig 2:**
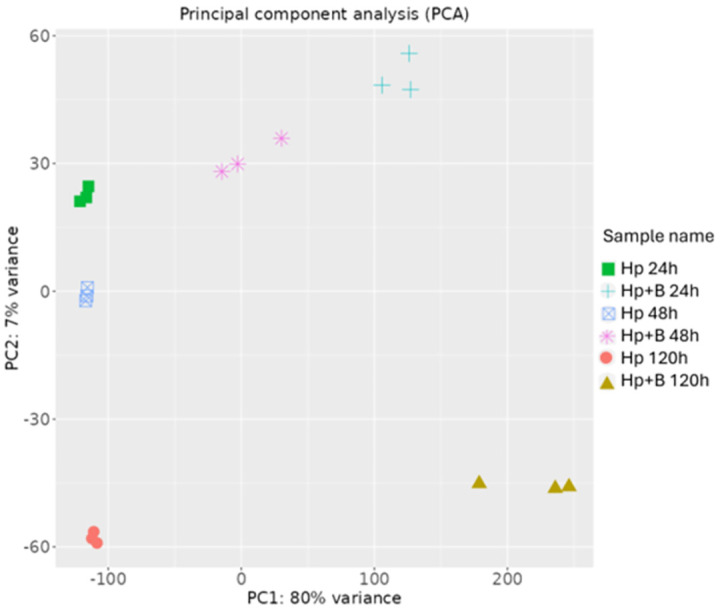
Principal Component Analysis (PCA) plot of the biological replicates of transcripts from *H*. *pseudoburtonii* (Hp) monocultures and *H*. *pseudoburtonii* and *B*. *cinerea* mixed cultures (Hp+B).

The expression levels of genes were determined by mapping sequence reads to the reference genome (genome and concatenated genome) of *H*. *burtonii* obtained from NCBI (GCF_001661395.1 and GCF_001661255.1). After trimming and filtering, more than 97% of high-quality reads (reads without adapters and low-quality bases, as well as artifacts or errors) per sample remained (S1 Table), which were used for subsequent analyses. In the co-culture of *H*. *pseudoburtonii* and *B*. *cinerea* after 24 and 48 h, less than 2% of the reads mapped to the *B*. *cinerea* genome, while after 120 h, just of over 5% of the reads mapped to the *B*. *cinerea* genome. These data suggest an overall suppression and growth retardation of *B*. *cinerea* in co-culture with *H*. *pseudoburtonii*. Principal Component Analysis (PCA) conducted on normalized data, showed that the biological replicates clustered together, indicating high reproducibility among samples. The first two principal components explained most of the variance: PC1 explained 80% of the variance and allowed for clear separation between monocultures and co-cultures, while PC2 which separated the three time points, explained 7% of the variance (Fig 2).

### Differential expression analysis

**Fig 3:**
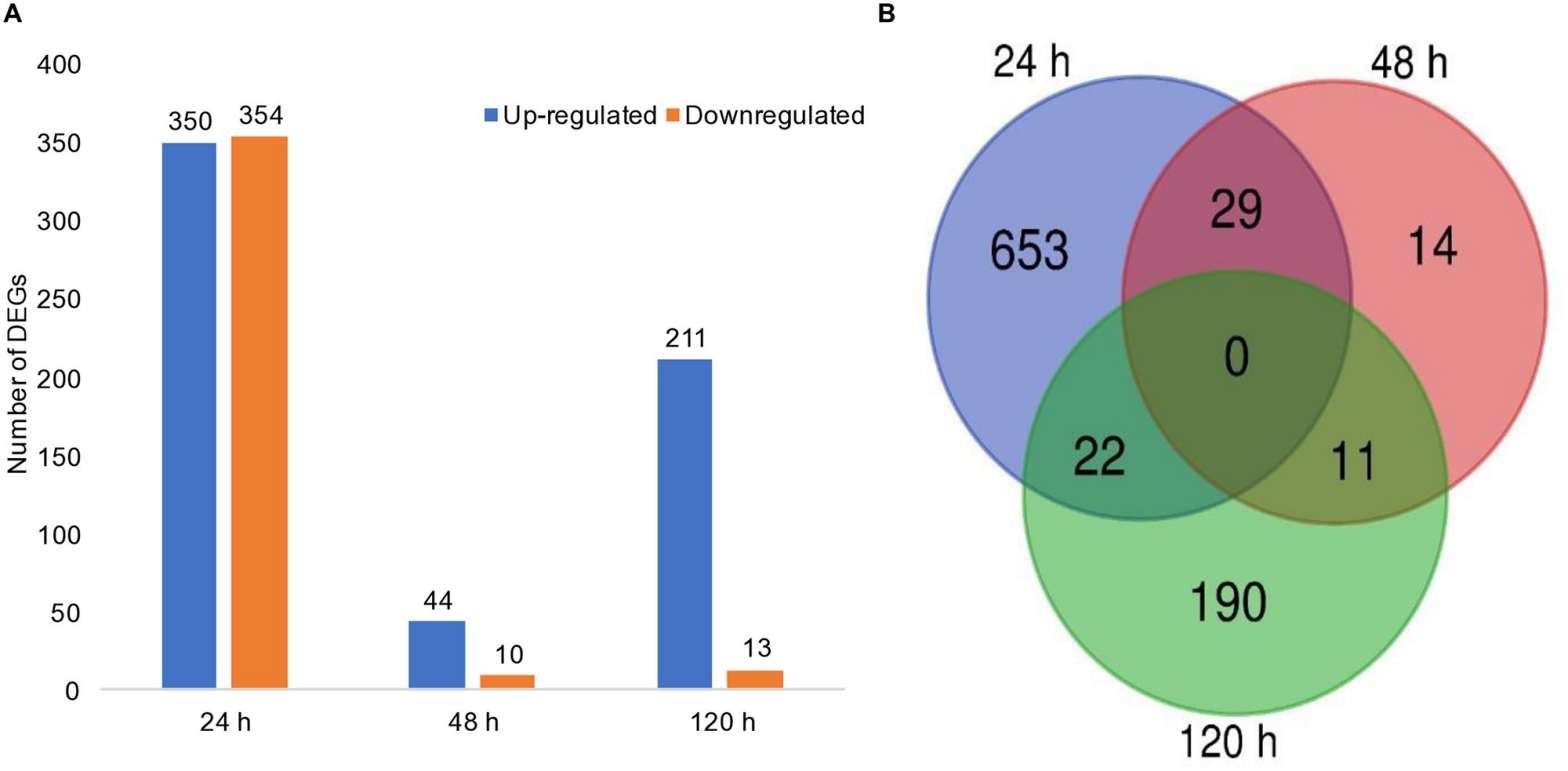
Representation of differentially expressed genes (DEGs) in *H*. *pseudoburtonii* at different time points (24, 48, and 120 h) in the presence of *B*. *cinerea* FF1. (A) Total number of up-regulated and down-regulated genes in *H*. *pseudoburtonii* in the co-culture. (B) Venn diagram showing the number of unique and shared DEGs between the different time points.

Differentially Expressed Genes (DEGs) were calculated with co-culture considered as the treatment group and monoculture serving as the reference baseline. A total of 919 differentially expressed genes (log2FC ≥ 1) were detected at the three time points. The highest number of DEGs was observed at 24 h, with 350 up-regulated and 354 down-regulated genes. At 48 and 120 h, there were a total of 54 DEGs (44 up-regulated and 10 downregulated) and 224 DEGs (211 up-regulated and13 downregulated), respectively (Fig 3A). A total of 29 DEGS were common between the 24 and 48 h sampling points, 11 between 48 and 120 h, and 22 between 120 h and 24 h (Fig 3B).

**Fig 4:**
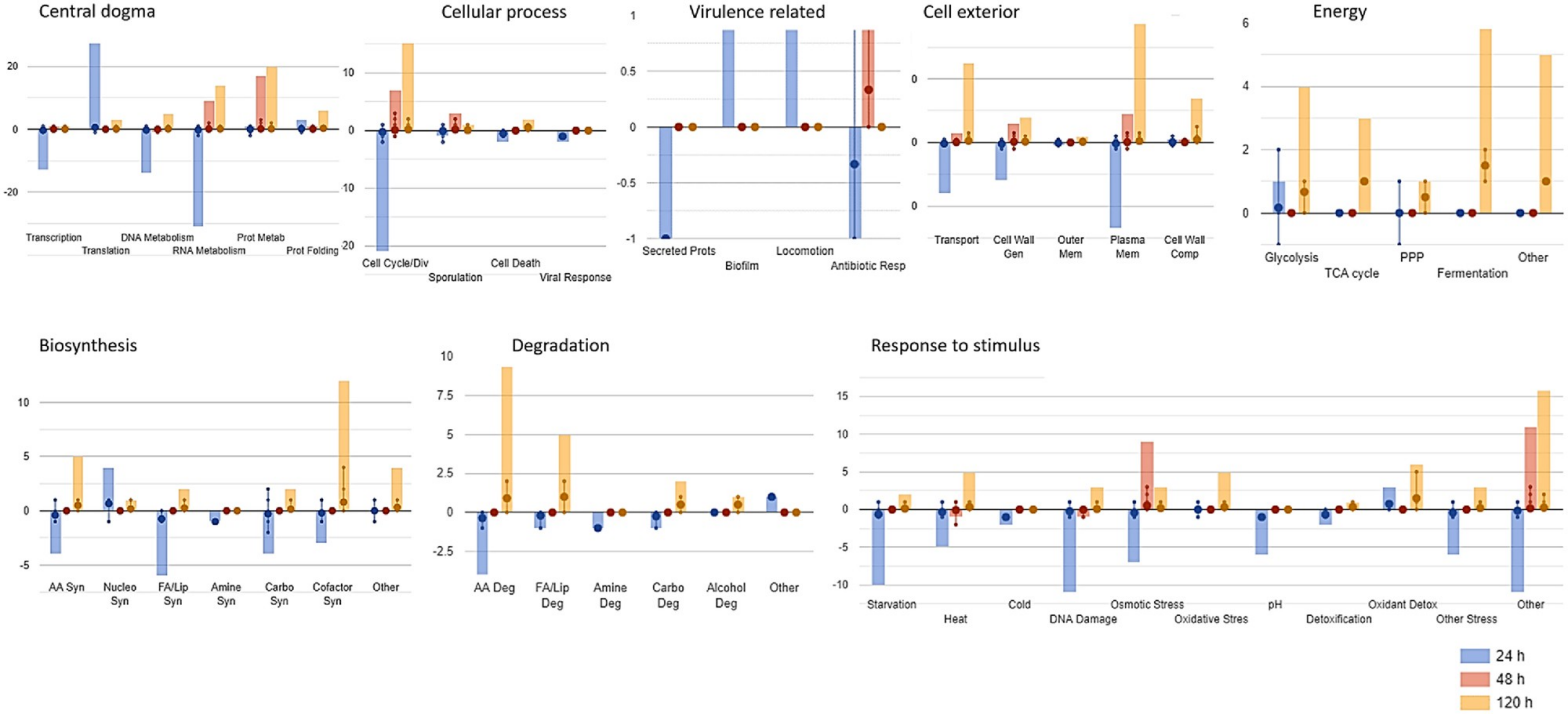
Subsystems associated with differentially expressed genes detected at 24, 48 and 120 h, in *H*. *pseudoburtonii* co-cultured with *B*. *cinerea* compared to *H*. *pseudoburtonii* monoculture. Each dot displayed on the vertical line represents the expression value for the genes. Whiskers, associated with box-and-whisker plots, indicate data range and variability. Bars represent averages or central tendencies. The Y-axis scales between the panels are different, impacting the comparability of values across the graph.

Differentially expressed genes (log2FC ≥ 1) were visualised on the Pathway Tools Omics Dashboard [47] to get an overview of *H*. *pseudoburtonii* transcriptome response patterns over time in presence of *B*. *cinerea*. Overall, the data show that at 24 h most subsystems were down-regulated, with only nucleotide synthesis, translation proteins and to a lesser extent glycolysis being up-regulated in the co-culture compared to the monoculture, while at 48 h RNA and protein metabolism as well as cell cycle/division and response to osmotic stress were up-regulated (Fig 4). At 120 h *H*. *pseudoburtonii* cells in co-culture display an overall up-regulation of energy metabolism, biosynthesis of amino acids and cofactors, cell cycle/division as well as amino acid and fatty acids degradation (Fig 4). At 24 h, the top 15 up-regulated genes mainly encode proteins involved in chromatin assembly and chromosome function (e.g., Histone H2A, H3, H4), and those involved in translation (e.g., *RPL22A*, *RPL43A* and *RPL36A*). Although not among the top 15, many genes encoding the 40S cytosolic small ribosomal subunit were up-regulated (S2 Fig). Conversely, the most down-regulated genes at this time point included several hypothetical proteins with unknown functions, as well as genes involved in cell wall integrity (*FKS1*), ethanol tolerance (*ETP1*), and RNA processing (*RRP5*) (S2 Table). At 48 h, genes mainly involved in cell cycle regulation and pseudohyphal growth were the most up-regulated (Table 1), while genes related to DNA repair and telomere maintenance (*RAD50*, *TEL1*), thiamine metabolism (*THI13*), and cell wall integrity (*WSC3*) were among the most down-regulated (S2 Table). In contrast, genes involved in the biosynthesis of thiamine and biotin, as well as the transport of zinc (*ZRT2*), copper (*CTR3*) and siderophore-iron (*FIT3)* were amongst the most up-regulated at 120 h (Table 1). At this time point, the most down-regulated genes were associated with cell wall organization (*DFG5*, *SCW4*), septin function (*SHS1*), and mitochondrial processes (*MRPL16*, *OAC1)* (S2 Table).

**Table 1:** Log2 fold change of the top 15 up-regulated genes in *H*. *pseudoburtonii* co-cultured with *B*. *cinerea* at 24, 48 and 120 h compared to the monoculture.

Time	Gene name	Description/Function	Log2FC
**24 h**	*HHF2*	Histone H4, DNA-binding subunit of the nuclear nucleosome	2.88
	*HTA1*	Histone H2A	1.56
	*HHT2*	Histone H3	1.52
	*RPL36A*	Ribosomal 60S subunit protein L36A	1.89
	*SDH5*	Protein required for flavinylation of Sdh1p	1.53
	*TMA10*	Protein of unknown function that associates with ribosomes	1.59
	*RPS17B*	Ribosomal protein 51 (rp51) of the small (40s) subunit	1.54
	*RAD1*	Single-stranded DNA endonuclease (with Rad10p)	1.54
	*RPS8B*	Protein component of the small (40S) ribosomal subunit	1.59
	*RPL22A*	Ribosomal 60S subunit protein L22A	1.58
	*RPP1A*	Ribosomal stalk protein P1 alpha	1.69
	*HTA2*	Histone H2A	1.54
	*YKL107W*	NADH-dependent aldehyde reductase	1.53
	*RPL34A*	Ribosomal 60S subunit protein L34A	1.79
	*INM1*	Inositol monophosphatase	1.56
**48 h**	*FLC2*	Flavin carrier protein	1.51
	*SNF5*	Subunit of the SWI/SNF	1.56
	*CDC39*	Regulator of transcription subunit	1.71
	*DHH1*	Cytoplasmic DEAD-box helicase	1.50
	*WHI4*	Protein WHI4; Putative RNA binding	2.48
	*LRG1*	Rho-GTPase-activating protein LRG1	2.87
	*STE5*	Pheromone-responsive MAPK scaffold	3.28
	*RTT103*	Regulator of Ty1 transposition protein	2.88
	*HKR1*	Signalling mucin HKR1	3.68
	*PUF4*	PUmilio-homology domain family	1.92
	*MAD1*	Coiled-coil protein	1.99
	*SLN1*	Osmosensing histidine protein kinase SLN1	2.87
	*PBS2*	MAP kinase of the HOG signaling pathway	1.58
	*CDC25*	Cell division control protein	1.69
	*TRF5*	Non-canonical poly(A) polymerase	2.25
	*SSK2*	MAP kinase kinase	2.48
	*PUF2*	PUmilio-homology domain Family	2.32
**120 h**	*SOD2*	Mitochondrial manganese superoxide dismutase	5.15
	*FIT3*	Mannoprotein involved in the retention of siderophore-iron in the cell wall	5.13
	*FOX2*	Fatty acid oxidation	2.30
	*KCC4*	Protein kinase of the bud neck involved in the septin checkpoint	2.80
	*PMA2*	Plasma membrane H+-ATPase	2.22
	*THI4*	Thiazole synthase	4.50
	*BIO2*	Biotin synthase	2.76
	*SCW11*	Putative glucanase	2.23
	*CTR3*	High-affinity copper transporter of the plasma membrane	3.40
	*AIM44*	Regulator of Cdc42p and Rho1p	2.32
	*ZRT2*	Low-affinity zinc transporter of the plasma membrane	3.03
	*HHF2*	Histone H4	2.45
	*PDC5*	Minor isoform of pyruvate decarboxylase	2.17
	*THI13*	Thiamine metabolism	3.08
	*HHT2*	Histone H3	2.37

**Fig 5:**
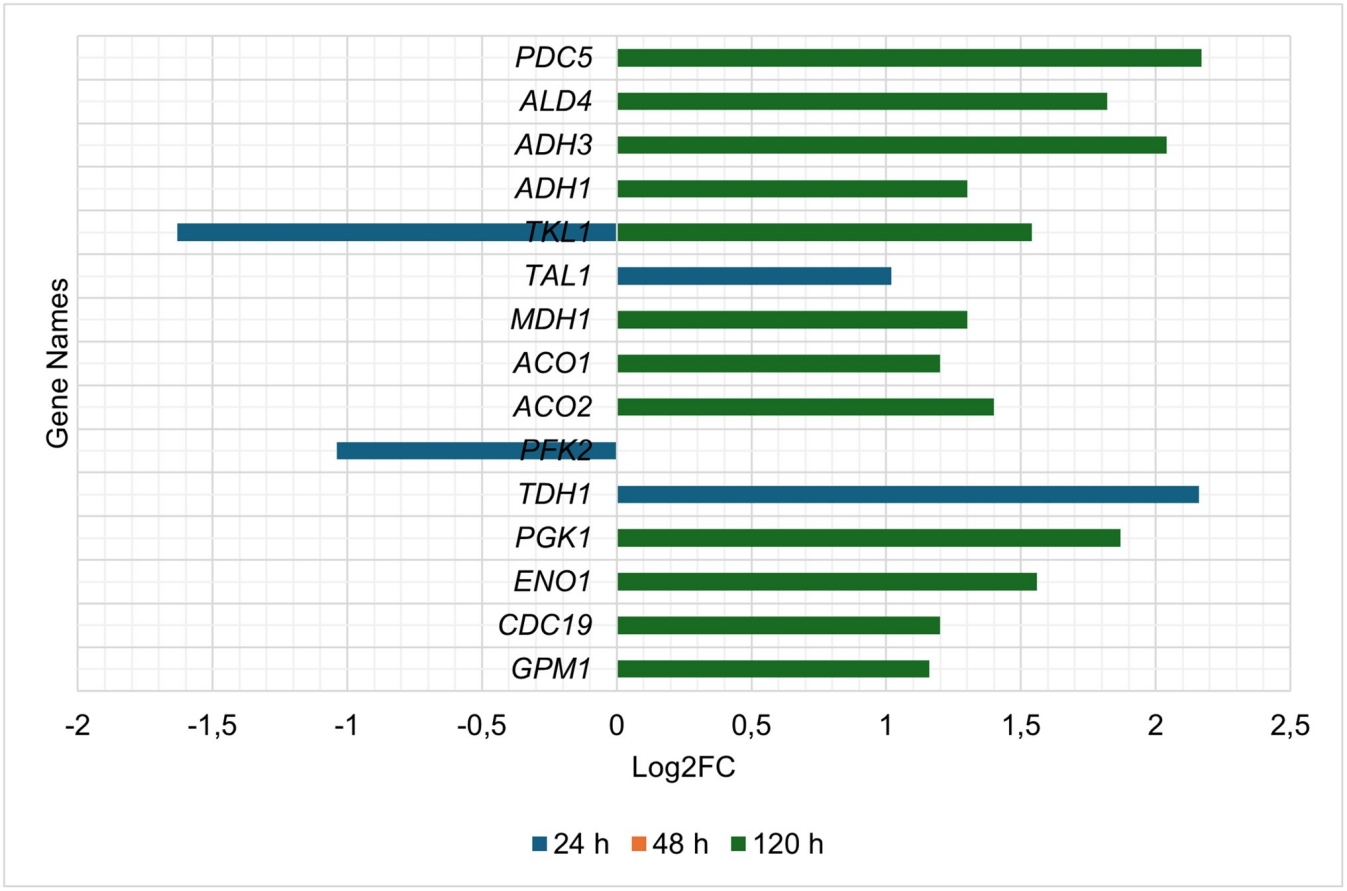
Energy metabolism-associated differentially expressed genes detected at 24, 48 and 120 h, in *H*. *pseudoburtonii* co-cultured with *B*. *cinerea* compared to *H*. *pseudoburtonii* monoculture.

Analysis of the energy metabolism subsystem revealed that multiple genes encoding enzymes in glycolysis, the tricarboxylic acid cycle (TCA) and glucose fermentation were significantly up-regulated at 120 h (Fig 5). These included genes such as *GPM1* (phosphoglycerate mutase), *CDC19* (pyruvate kinase), *ENO1* (phosphopyruvate hydratase), and *PGK1* (phosphoglycerate kinase) in glycolysis, *ACO2* (aconitate hydratase), *ACO1* (aconitase), and *MDH1* (malate dehydrogenase) in the TCA cycle, and *ADH1* (alcohol dehydrogenase 1), *ADH3* (alcohol dehydrogenase 3), *ALD4* (aldehyde dehydrogenase), and *PDC5* (pyruvate decarboxylase 5) involved in fermentation.

### GO enrichment analysis of DEGs in *H*. *pseudoburtonii* co-cultured with *B*. *cinerea*

**Fig 6:**
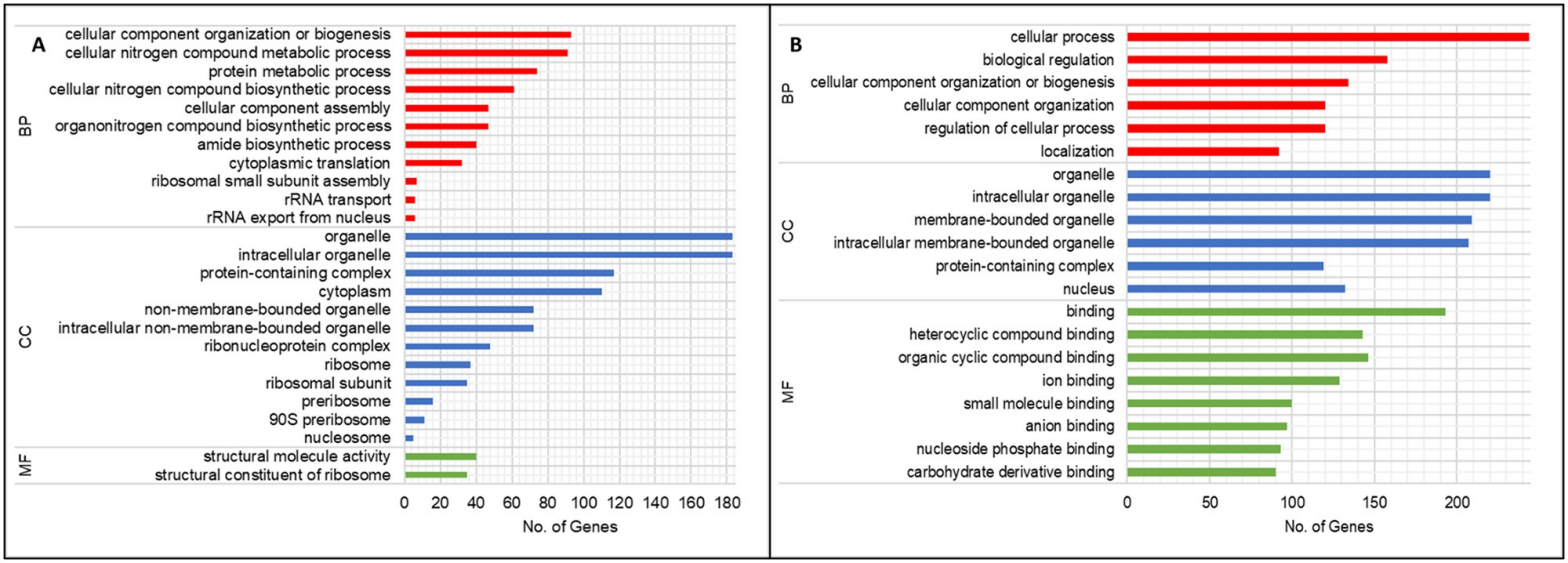
Gene ontology classification of up-regulated (A) and down-regulated (B) genes (Log2FC ≥1, p- value < 0.05) in *H*. *pseudoburtonii* co- cultured with *B*. *cinerea* compared to the monoculture at 24 h, showing enriched biological process (BP), cellular component (CC) and molecular function (MF).

Gene ontology enrichment was used in the current study to further analyse the functions of the DEGs. Overall, genes that were significantly up-regulated in *H*. *pseudoburtonii* when co-cultured with *B*. *cinerea* compared to monoculture, after 24, 48 and 120 h were functionally classified into the major gene ontology categories: biological process (BP), molecular function (MF) and cellular component (CC). Conversely, due to a limited number of significantly down-regulated genes, at 48 and 120 h, functional classification of these DEGs into the relevant categories was not achieved. Based on the GO enrichment analysis of the up-regulated genes, the significantly enriched biological processes after 24 h (Fig 6A) were related to protein translation processes such as cellular component organization or biogenesis (GO:0071840), cellular nitrogen compound metabolic process (GO:0034641), protein metabolic process (GO:0019538) and cellular nitrogen compound biosynthetic process (GO:0044271). The enriched molecular functions were structural constituent of ribosome (GO:0003735) and structural molecule activity (GO:0005198), while the organelle (GO:0043226), intracellular organelle (GO:0043229), protein-containing complex (GO:0032991) and cytoplasm (GO:0005737) were the most enriched cellular components. The genes associated with the ribosome were almost exclusively up-regulated (S3 Table). Conversely, cellular process (GO:0009987) and biological regulation (GO:0065007) were the most enriched biological processes with down-regulated genes (Fig 6B). Binding (GO:0005488) of various types of compounds was enriched as significantly down-regulated molecular function, with genes associated with these functions mainly localised in various intracellular organelle (GO:0043229), the nucleus (GO:0005634) and protein-containing complex (GO:0032991).

**Fig 7:**
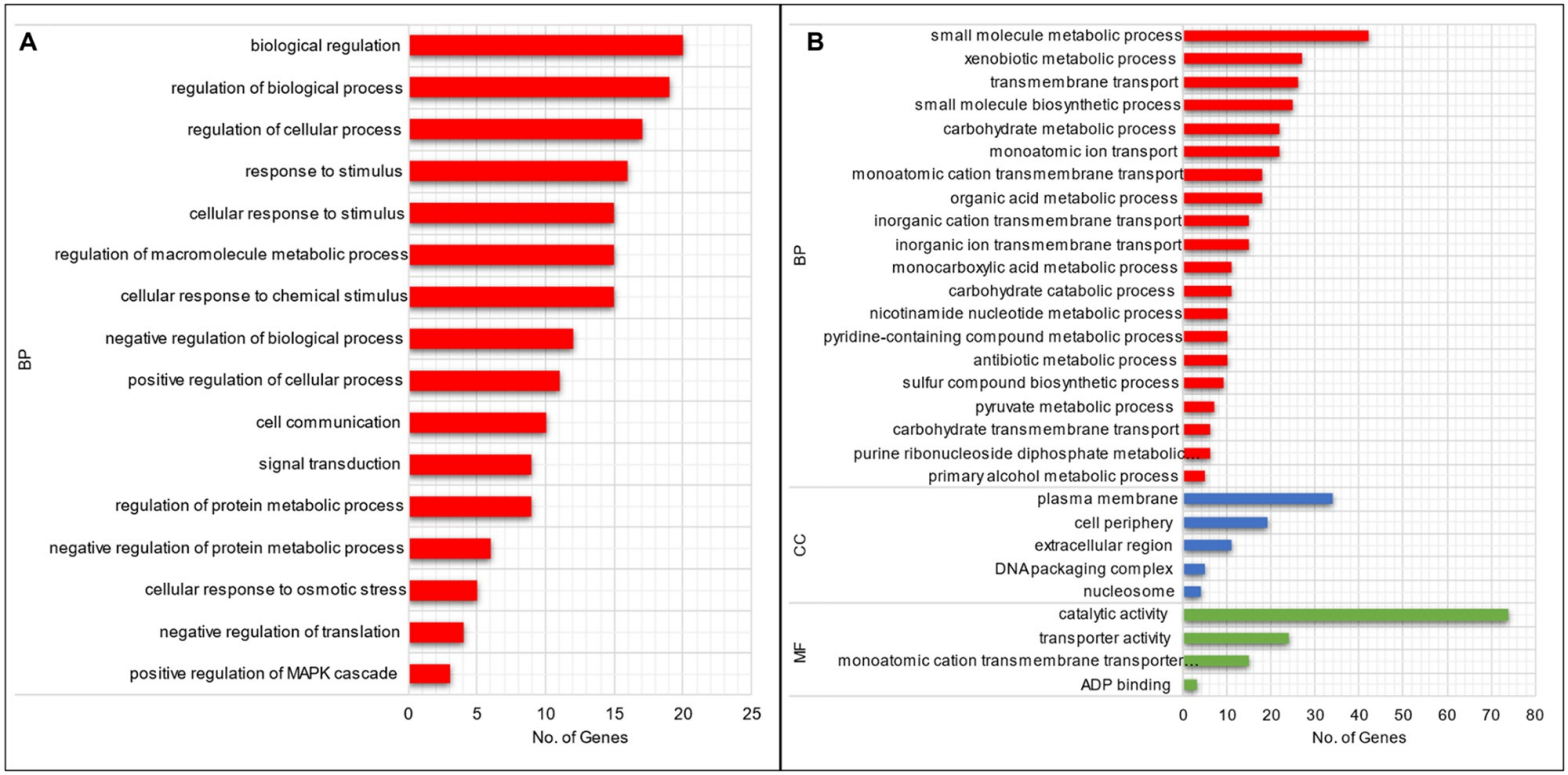
Gene ontology classification of up-regulated DEGs in *H*. *pseudoburtonii* grown for (A) 48 h and (B) 120 h in the presence of *B*. *cinerea*, showing enriched biological process (BP), cellular component (CC) and molecular function (MF).

After 48 h of co-cultivation, GO analysis of up-regulated genes of *H*. *pseudoburtonii* revealed an enrichment of biological processes (Fig 7A) associated with regulation of various biological processes (GO:0065007) and cellular processes (GO:0050794), as well as response to stimuli (GO:0050896), compared to the monoculture. No significant enrichment occurred for molecular function or cellular component.

**Fig 8:**
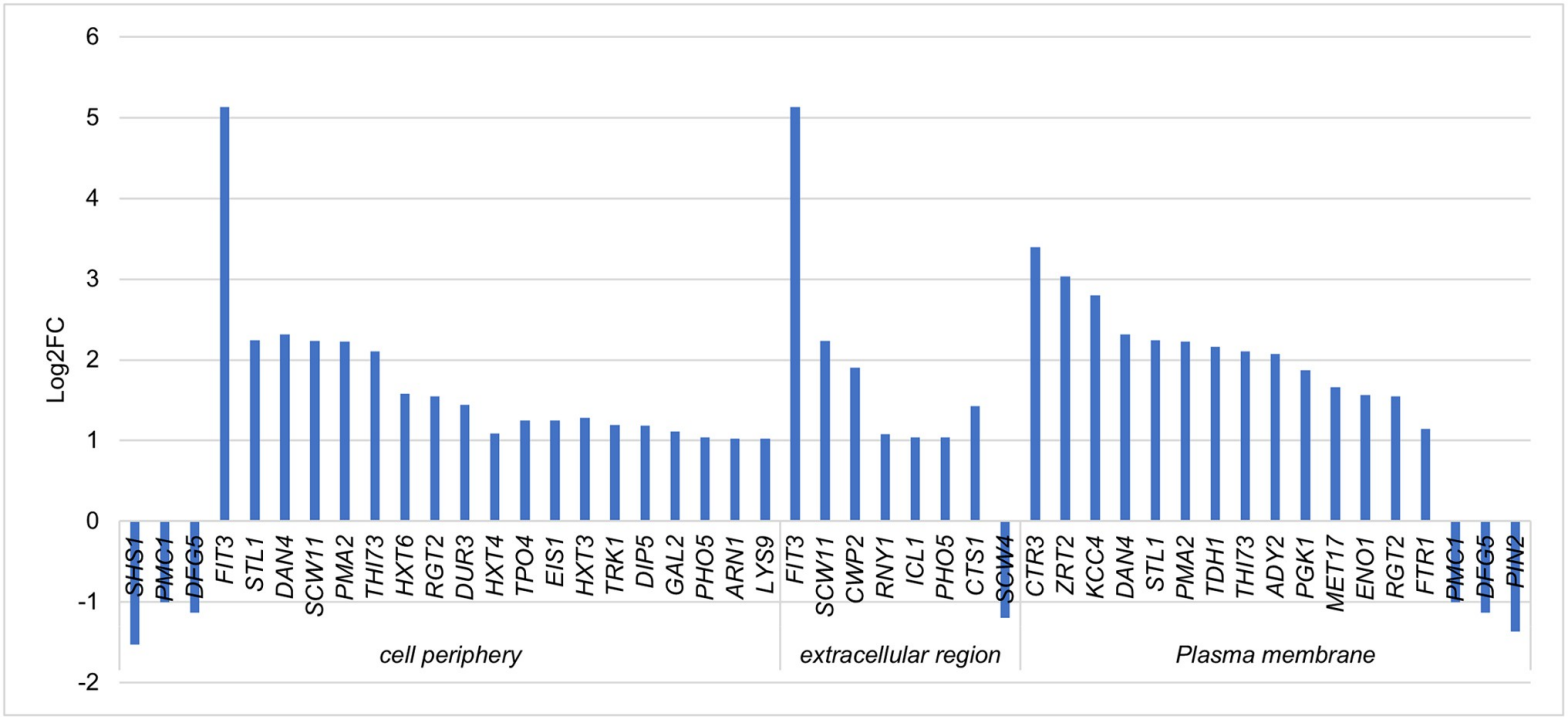
Up-regulated and down-regulated genes associated with the enrichment of the cellular component GO term: Cell periphery, extracellular region and plasma membrane of *H*. *pseudoburtonii* co-cultured with *B*. *cinerea* at 120 h.

When considering the DEGs in *H*. *pseudoburtonii* when in co-culture with *B*. *cinerea* compared to the monoculture at 120 h, GO enrichment highlighted small molecule biosynthetic process (GO:0044283), small molecule metabolic process (GO:0044281), xenobiotic metabolic process (GO:0006805) and transmembrane transport (GO:0055085) as the most enriched up-regulated biological process. Catalytic activity (GO:0003824) and transporter activity (GO:0005215) were the most up-regulated molecular function. Additionally, plasma membrane (GO:0005886) and cell periphery (GO:0071944) were the most enriched up-regulated cellular component, respectively, (Fig 7B). Our data also revealed transporter activity as an enriched molecular function notably genes encoding zinc transporters *ZRT2*, sugar transporters *STL1*, *GAL2*, *RGT2*, *HXT4*, *HXT6*, *HXT3*; copper transporters *CTR2*, *CTR3* and iron transporters *FIT3*, *ARN1*, *FTR1*, were significantly up-regulated (Fig 8).

### KEGG pathways enrichment analysis in *H*. *pseudoburtonii* co-cultured with *B*. *cinerea*

**Fig 9:**
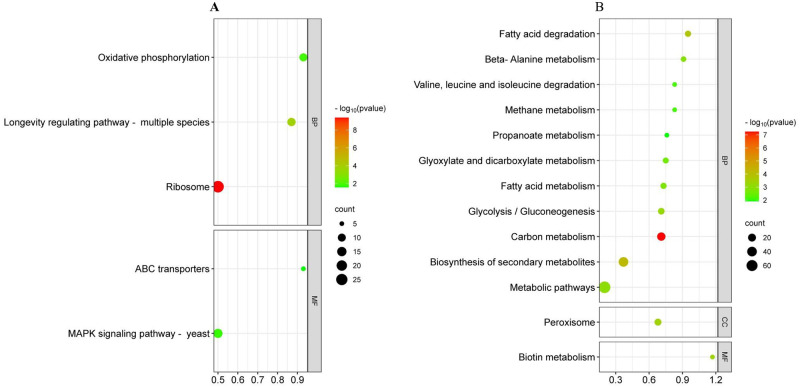
KEGG pathways of down and up- regulated genes in *H*. *pseudoburtonii* grown for (A) 24 and (B) 120 h in the presence of *B*. *cinerea*.

The KEGG enrichment analysis of differentially expressed genes (DEGs) in *H*. *pseudoburtonii* grown for 24, 48, and 120 h in the presence of *B*. *cinerea* revealed that 19 KEGG pathways were significantly enriched (p ≤ 0.05). The ribosome and oxidative phosphorylation pathways were significantly enriched at 24 h, with 22 and 17% of the genes associated with these pathways, respectively, up-regulated, while the down-regulated genes exhibited significant associations with the MAPK signalling pathway, ABC transporters, and the longevity regulation pathway. Conversely, the MAPK signalling pathway was enriched with the up-regulated genes at 48 h. Pathways enriched at 120 h primarily involve carbohydrate, lipid, and amino acid processing, alongside the breakdown and utilization of external substances. Enriched KEGG pathways at 120 h included carbon metabolism, biosynthesis of secondary metabolites, glycolysis/gluconeogenesis, glyoxylate and dicarboxylate metabolism, and biosynthesis of amino acids, among others (Fig 9 and S3 Table).

### Competition for iron

The role of competition for iron on the antagonistic activity of *H*. *pseudoburtonii* against *B*. *cinerea* was investigated on solid and liquid media. On solid medium the development of a reddish halo around the colony on PDA supplemented with FeCl_3_ was investigated and the width of the mycelial inhibition zone was also measured. In this study, no reddish halo was observed and the level on mycelial inhibition was similar at across the different concentration of FeCl_3_ and at both pH 4.5 and 5.6 (S3 Fig). In addition, no hydroxamate-type siderophores could be detected in the supernatants of *H*. *pseudoburtonii* as well as co-cultures with *B*. *cinerea* in malt extract broth at different time points.

### Global analysis of exo-proteome

Along with transcriptome analysis, the exo-proteome of *H*. *pseudoburtonii* (Hp) under both monoculture and co-culture conditions with *Botrytis cinerea* (Hp + B) for 48 h were analyzed. A total of 85 proteins were identified, with 78 proteins being common to both Hp and Hp+B (S3 Table). The identified proteins were categorized into distinct functional clusters, as detailed in S4 Table. Noteworthy clusters included the 40S ribosomal protein S1, 60S ribosomal protein L8, cell wall protein PhiA, translation elongation factor 1-alpha (fragment), phosphopyruvate hydratase, and glyceraldehyde-3-phosphate dehydrogenase, among others.

Proteins that were exclusively present in the co-culture (Hp+B) or exhibited a fold change (FC) ≥ 1.2 were classified as differentially abundant proteins (DAPs). A total of 22 DAPs (unique and differentially abundant proteins) were identified. The seven unique proteins were elongation factor 2, 40S ribosomal protein S16, glycoside hydrolase from the glycosyl hydrolase family 17 (A0A1E4RPI3_9ASCO), phosphoglucomutase (α-D-glucose-1,6-bisphosphate-dependent), heat shock protein 70, and two isoforms of elongation factor 1-α (Table 2). The most abundant proteins identified included alpha-1,4 glucan phosphorylase, pyruvate kinase, ATP synthase subunit alpha, protein TOS1, and ribosomal protein S4 (Table 2).

**Table 2:** Unique and differentially abundant proteins identified in *H*. *pseudoburtonii* + *B*. *cinerea* (Hp+B) co-cultures.

	Category	Identified Proteins	Accession Number	*p*-value	FC
**Unique proteins**	**Protein folding and stress response**	Heat shock protein 70	A0A1E4RQG3_9ASCO	0,028	0,2
	**Carbohydrate metabolism:**	Glycoside hydrolase	A0A1E4RPI3_9ASCO	0,011	0,5
		Phosphoglucomutase (alpha-D-glucose-1,6-bisphosphate-dependent)	A0A1E4RCR6_9ASCO	0,049	0,7
	**Protein synthesis and translation:**	40S ribosomal protein S16	A0A1E4RR23_9ASCO	0,013	0,3
		Elongation factor 2	A0A1E4RI69_9ASCO	0,025	0,3
		Elongation factor 1-α (Fragment)	A0A1B0Z732_9ASCO	0,016	0,8
		Elongation factor 1-α (Fragment)	A0A0D3RPB1_9ASCO	0,049	0,8
**DAPs**	**Protein synthesis and translation:**	40S ribosomal protein S1	A0A1E4RR15_9ASCO	0.61	1.8
		Ribosomal protein S4	A0A1E4RPW6_9ASCO	0.33	4.7
		40S ribosomal protein S14	A0A1E4RFC9_9ASCO	0.41	1.6
	**Glycolysis and energy metabolism:**	D-xylose reductase (NAD(P)H)	A0A1E4RLY1_9ASCO	0.75	1.2
		Phosphoglycerate mutase	A0A1E4RM70_9ASCO	0.59	1.2
		Six-hairpin glycosidase (Fragment)	A0A1E4RGA6_9ASCO	0.81	1.2
		DUF3757 domain-containing protein	A0A1E4REI4_9ASCO	0.62	1.3
		Plasma membrane ATPase	A0A1E4RI84_9ASCO	0.66	1.5
		Chaperonin GroL	A0A1E4RHK0_9ASCO	0.69	1.6
		Transaldolase	A0A1E4REE9_9ASCO	0.61	1.6
		Alpha-1,4 glucan phosphorylase	A0A1E4RED5_9ASCO	0.4	2.2
		Pyruvate kinase	A0A1E4RTD9_9ASCO	0.25	2.3
		ATP synthase subunit alpha	A0A1E4RJW3_9ASCO	0.35	2.3
	**Cell wall-related**	Protein TOS1	A0A1E4RQ57_9ASCO	0.059	3.3
		PR-1-like protein	A0A1E4RCA1_9ASCO	0.5	1.2

## Discussion

The current study used RNA sequencing to assess the temporal changes in the transcriptome of *H*. *pseudoburtonii* in co-culture with *B*. *cinerea* and decipher potential mechanisms underlying the antifungal activity in this yeast. Initial analysis of the sequencing reads provides compelling evidence of the presence of *B*. *cinerea*, exhibiting a gradual increase in mapping reads from 1% to 5%. Nonetheless, the amount of reads mapping to the *B*. *cinerea* genome were extremely low, indicating the inhibitory effects exerted by *H*. *pseudoburtonii* on *B*. *cinerea*. Evaluation of differentially expressed genes revealed an activation of genes associated with the major core histones, and ribosomal activity and down-regulation of binding activity in the first 24 h. The up-regulated activities mostly represented ribosome biogenesis, as well as oxidative phosphorylation. These were also accompanied by a down-regulation of genes associated with longevity, particularly glucose-repressible genes and genes that regulate growth and carbon utilization during nutrient limitation. Ribosome biogenesis is pre-requisite for the production of proteins which are indispensable for cell growth and proliferation [48]. We can infer from gene expression patterns, that in the first 24 h *H*. *pseudoburtonii* begins to adapt to its surrounding environment, does not experience any nutrient limitation and directs energy towards building capacity for protein synthesis cell growth and cell proliferation. Subsequently, an up-regulation of genes indicating cell cycle passage through START (e.g., *WHI4*, *LRG1*, *MAD1*, *CDC25*), as well as those associated with signalling pathways and cell communication processes (e.g., *SLN1*, *PBS2*, *SSK2*) and filamentous growth (e.g., *SNF5*, *CDC39*, *STE5*, *DHH1*) were up-regulated. The up-regulated genes involved in signalling are homologous to those encoding proteins in the high osmolarity growth MAP kinase cascade which displays a conserved role in osmoadaptation in fungi but has also been shown to have additional biological functions [49, 50]. For instance, the Ssk2p/Pbs2p/Hog1p MAPK cascade has been shown to regulate yeast-to-hyphae transition in *S*. *cerevisiae* [51], *Candida albicans* [52] and *Yarrowia lipolytica* [53], hyphal growth, branching and plant infection in *Fusarium graminearum* [54], as well as conidiation and trap morphogenesis in the nematode trapping fungus, *Arthrobotrys oligospora* [55]. Moreover, the MAPK cascades have been shown to play a central role in the regulation of mycoparasitic activity of *Trichoderma* spp. against phytopathogens such as *B*. *cinerea* and *Rhizoctonia solani* [56, 57]. The transcriptome dynamics in the first 48 h suggest that the interaction between *H*. *pseudoburtonii* and *B*. *cinerea* is characterized by an initial adaptation and response to external stimuli, as well as active growth of *H*. *pseudoburtonii* resulting in the formation of hyphae. This is further supported by an enrichment in energy metabolism seen in glycolytic processes and the pentose phosphate pathway, along with a notable increase in oxidative phosphorylation pathway which supports enhanced ATP synthesis for cellular activities of yeast cells [58].

During the later stages (120 h), several genes including *PMC1*, *DFG5*, *SCW4*, *SHS1*, and *PIN2* were downregulated in *H*. *pseudoburtonii* when co-cultured with *B*. *cinerea*. The downregulation of *PMC1*, which encodes a vacuolar Ca2+ ATPase, suggests potential alterations in calcium homeostasis within the yeast cells. This change may reflect broader modifications in signalling pathways or stress response mechanisms [59]. The downregulation of *SHS1* and *PIN2*, genes involved in cell division and cycle progression, indicates a potential slowdown in *H*. *pseudoburtonii’s* growth and proliferation in the presence of *B*. *cinerea*. This could represent a shift in resource allocation from growth to defence or stress response mechanisms. Additionally, the observed downregulation of *SCW4* and *DFG5*, which are involved in cell wall maintenance and remodelling, could indicate potential alterations in *H*. *pseudoburtonii’s* cell wall structure or integrity. This change may be an adaptive response to stress induced by the presence of *B*. *cinerea* [60, 61]. Several genes involved in cell wall biogenesis and stability, including *CTS1*, *SCW11*, *CWP2* and *DAN4*, were up-regulated during the later stages (120 h). These genes have specific functions: *CTS1* encodes an endochitinase, while *SCW11* codes for a glucanase, and *CCP2* and *DAN4* code for cell wall mannoproteins. Both *CTS1* and *SCW11* are transcribed in early G_1_ and expressed only in daughter cells; they are required for cytokinesis and cell separation [62, 63]. *KCC4* encoding a protein kinase involved in morphogenesis checkpoint and budding cell bud growth was among the top 15 up-regulated genes. The data also revealed significant up-regulation of genes involved in nutrient acquisition and oxidative stress tolerance. In particular, gluconeogenesis, the glyoxylate cycle and fatty acid metabolism were enriched along with biotin and thiamine biosynthesis. Biotin is a pivotal cofactor for many enzymes involved in gluconeogenesis, lipid biosynthesis and amino acid metabolisms [64]. These data highlight activation of alternative carbon metabolism in *H*. *pseudoburtonii*. An enrichment of peroxisomes was apparent in the 120 h transcriptome. This aligns with the up-regulation of the lipid metabolism and reflects an ability of the yeast to detoxify toxic reactive oxygen species (ROS) through the activity of catalase (encoded by *CTA1*, log2FC = 1.1) and superoxide dismutase (encoded by *SOD2*, log2FC = 5.2), both of which were significantly up-regulated. Taken together, the data show transcriptome response akin to cells entering quiescence. Indeed, studies have shown that quiescent yeast cells up-regulate genes required for respiration, glyoxylate cycle, fatty acid metabolism as well as antioxidant defences to maintain low ROS [65]. In the current study, it was evident that *H*. *pseudoburtonii* cells grew rapidly in the first 24 h and remained in stationary phase for a long period after that. Stationary phase yeast culture comprises a heterogeneous population of quiescent and non-quiescent cells [66]. Quiescence would have been triggered by nutrient depletion, which can be expected in a cell population that would had been growing in malt extract broth for 120 h.

Under the prevailing condition, gene expression patterns suggested competition for iron as a possible mechanism of interaction between *H*. *pseudoburtonii* and *B*. *cinerea*. Indeed, several genes that encode proteins facilitating the uptake of different sources of iron were significantly up-regulated. These include Fit3p which facilitates the retention of siderophore-iron chelates in the cell wall, Arn1p which transports ferrichrome and hydroxamates of the ferrichrome type, and Sit1p which recognizes a wide variety of ferrichromes and coprogen as well as the Fet3p/Ftr1p oxidase/permease high affinity complex which supports yeast growth in low iron environments. However, in the current study no obvious iron chelation or production of hydroxamate-type siderophores could be detected in *H*. *pseudoburtonii* when grown alone or co-cultured with *B*. *cinerea* on agar or in broth. Therefore, the data suggest that unlike some yeasts such as *Methschinowia pulcherrima*, *Metschnikowia fruticola*, *Rhodotorula glutinis* as well as the yeast-like fungus, *Aureobasidium pullulans*, that chelate iron through the production of siderophores, thereby making it unavailable for phytopathogens [67, 68], *H*. *pseudoburtonii* does not secrete siderophores. Rather, the upregulation of the genes encoding proteins involved in the uptake of siderophore-bound iron could simply be a response to iron deprivation as has been observed in *Saccharomyces cerevisiae* [69].

Analysis of the exo-proteome of *H*. *pseudoburtonii* after 48 h of contact with *B*. *cinerea* was characterized by an abundance of proteins involved in carbohydrate metabolism, particularly in the pentose phosphate pathway (PPP), as well as proteins related to protein synthesis, ATP synthesis, and stress response. These enzymes are canonically intracellular proteins. However, some of them e.g., phosphoglycerate mutase, S14 ribosomal protein and subunits of ATP synthase have been shown to be moonlighting proteins. Such proteins have been shown to have a second function when outside the cell or bound to the cell surface and are used as adhesins to interact with host cells and proteins [70, 71]. Consequently, their abundance in the exo-proteome could suggest that they play a crucial role in the interaction between *H*. *pseudoburtonii* and *B*. *cinerea*. The genes encoding some of these proteins e.g., *ATP7* (log2FC 1.03) encoding ATP synthase, *ELF1* (log2FC 1.21) encoding elongation factor 1 as well as several genes encoding various components of the 40S ribosomal small subunit including *RPS1B* (log2FC 1.49), and *RPS14A* (log2FC 1.06) were up-regulated in the first 24 h which could explain the accumulation of the proteins at 48 h.

The proteomic data from *H*. *pseudoburtonii* co-cultivated with *B*. *cinerea* supernatants showed partial alignment with transcriptome data. Nonetheless, the two approaches both suggest that the antagonistic interaction between *H*. *pseudoburtonii* and *B*. *cinerea* potentially involves physical contact and adherence of *H*. *pseudoburtonii* to *B*. *cinerea* hyphae, as well as competition for nutrients, in particular iron. This convergence of findings from both transcriptomic and proteomic analyses provides a level of validation for the transcriptomic results. The competition for nutrient acquisition, especially iron, is a phenomenon observed in other microbial antagonism studies as well. While there were differences in the specific proteins and transcripts identified by each method, likely due to the different sensitivities and regulatory mechanisms influencing protein and mRNA levels, the overall biological implications remain consistent. This study represents a significant advancement in understanding the antagonistic mechanisms of *H*. *pseudoburtonii* against *B*. *cinerea*, highlighting its potential as a biocontrol agent against phytopathogens. While further *in vivo* studies and practical validation are needed, this research contributes valuable insights to scientific understanding and lays groundwork for potential agricultural and viticultural applications in sustainable vineyard management. While our transcriptomic and proteomic analysis provides valuable insights into the interaction between *H*. *pseudoburtonii* and *B*. *cinerea*, we acknowledge the need for further validation of these results. Future studies should focus on developing genetic tools for *H*. *pseudoburtonii* to enable mutant generation and more targeted functional analyses.

## Conclusion

The study findings reveal that *H*. *pseudoburtonii* employs a complex strategy in response to *B*. *cinerea*. The analysis provides important insights into gene expression patterns that shed light on the dynamics between *H*. *pseudoburtonii* and *B*. *cinerea*, particularly at specific time points. This newly discovered knowledge holds great potential for further characterizing *H*. *pseudoburtonii* as a potential and an effective biocontrol agent. By examining how *H*. *pseudoburtonii* responds to *B*. *cinerea* on a molecular level, the study establishes a solid foundation for unravelling the intricate biological mechanisms involved in successful biocontrol systems. The research provides valuable information about the molecular intricacies governing interactions between hosts and pathogens, offering guidance to improve our understanding and management of fungal interactions. Overall, this study contributes to the growing body of knowledge on biocontrol systems and provides a basis for future research in this area.

## Supporting information

S1 Fig Microscopic observation of *B*. *cinerea* FF1 and *H*. *pseudoburtonii* monocultures as well as their co-culture at 24, 48 and 120 h, images were captured at 400x magnification using a light microscopy (A) and viable cell count (CFU/mL) of *H*. *pseudoburtonii* in monoculture (Hp) and co-culture (Hp + B). The cell enumeration was performed on Wallerstein Nutrient Laboratory agar medium and grown at 25°C (B).
(TIF)

S2 Fig Central dogma-associated differentially expressed genes detected at 24 and 48 h in *H*. *pseudoburtonii* co-cultured with *B*. *cinerea* compared to *H*. *pseudoburtonii* monoculture. (TIF)

S3 Fig Effect of iron concentration on the antagonistic activity and mycelial growth inhibition of *Hyphopichia pseudoburtonii* Y963 against *Botrytis cinerea* IWBT-FF1 at pH 4.5 and pH 5.6 on potato dextrose agar. Different letters over the bars show significant differences between the iron concentrations according to Tukey’s post hoc test (p = 0.05).
(TIF)

S1 Table Overview of the number of raw, quality trimmed/filtered and mapped reads for the interaction experiment of *H*. *pseudoburtonii* Y963 with *B*. *cinerea* IWBT-FF1. (DOCX)

S2 Table Log2 fold change of the top 15 downregulated genes in *H*. *pseudoburtonii* co-cultured with *B*. *cinerea* at 24, 48 and 120 h compared to the monoculture. (DOCX)

S3 Table Significantly enriched KEGG pathways (q ≤ 0.05) of DEGs in *H*. *pseudoburtonii* grown for 24, 48 and 120 h in the presence of *B*. *cinerea*. (DOCX)

S4 Table Protein and clusters identified in *H*. *pseudoburtonii* monoculture and *H*. *pseudoburtonii* + *B*. *cinerea* co-cultures. T-Test (p-value) and fold change (FC) were calculated in SCAFFOLD software.
(DOCX)
