# Supplementary figures and images for: Unravelling the transcriptomic dynamics of Hyphopichia pseudoburtonii in co-culture with Botrytis cinerea
Source: PLoS One. 2025 Jan 14;20(1):e0316713. doi: 10.1371/journal.pone.0316713 (PMC11731708; doi:10.1371/journal.pone.0316713)

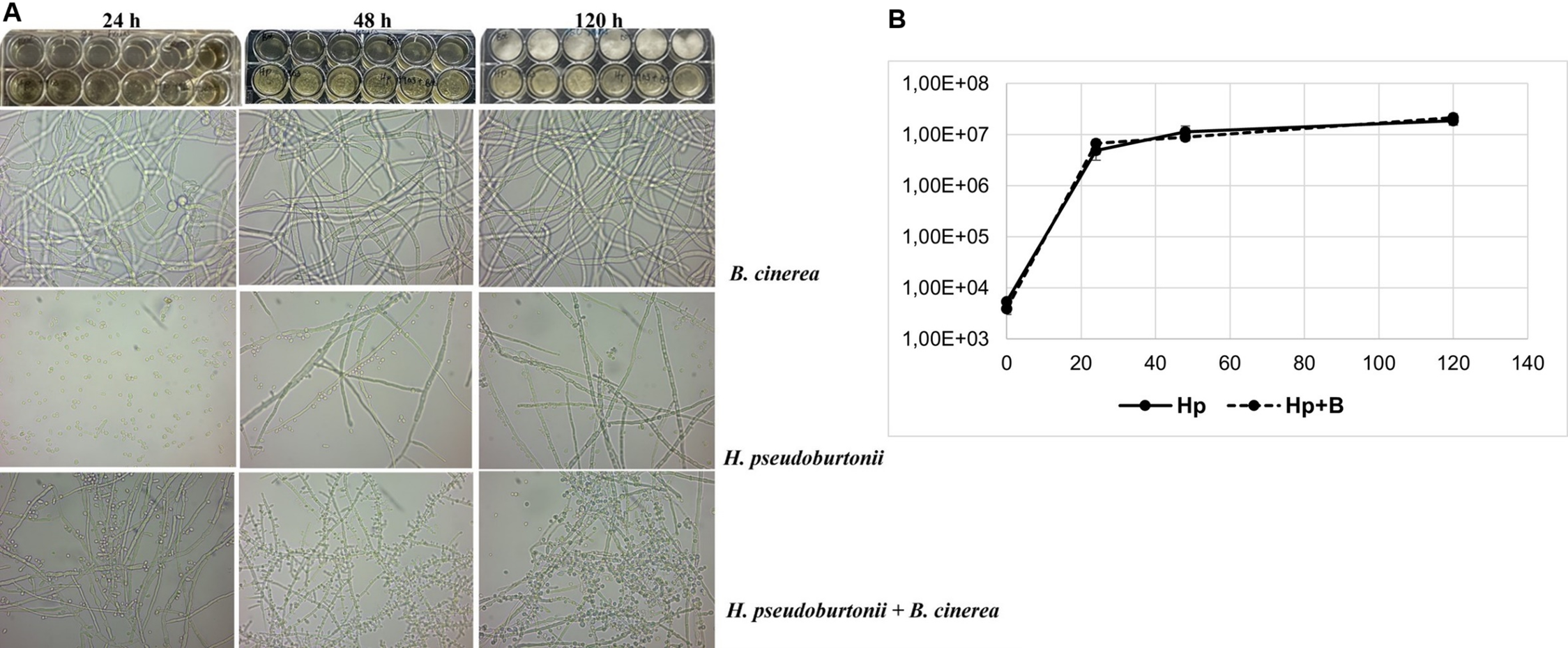

Supplement: S1 Fig — Microscopic observation of B. cinerea FF1 and H. pseudoburtonii monocultures as well as their co-culture at 24, 48 and 120 h, images were captured at 400x magnification using a light microscopy (A) and viable cell count (CFU/mL) of H. pseudoburtonii in monoculture (Hp) and co-culture (Hp + B). The cell enumeration was performed on Wallerstein Nutrient Laboratory agar medium and grown at 25°C (B). (TIF) [file pone.0316713.s001.tif]

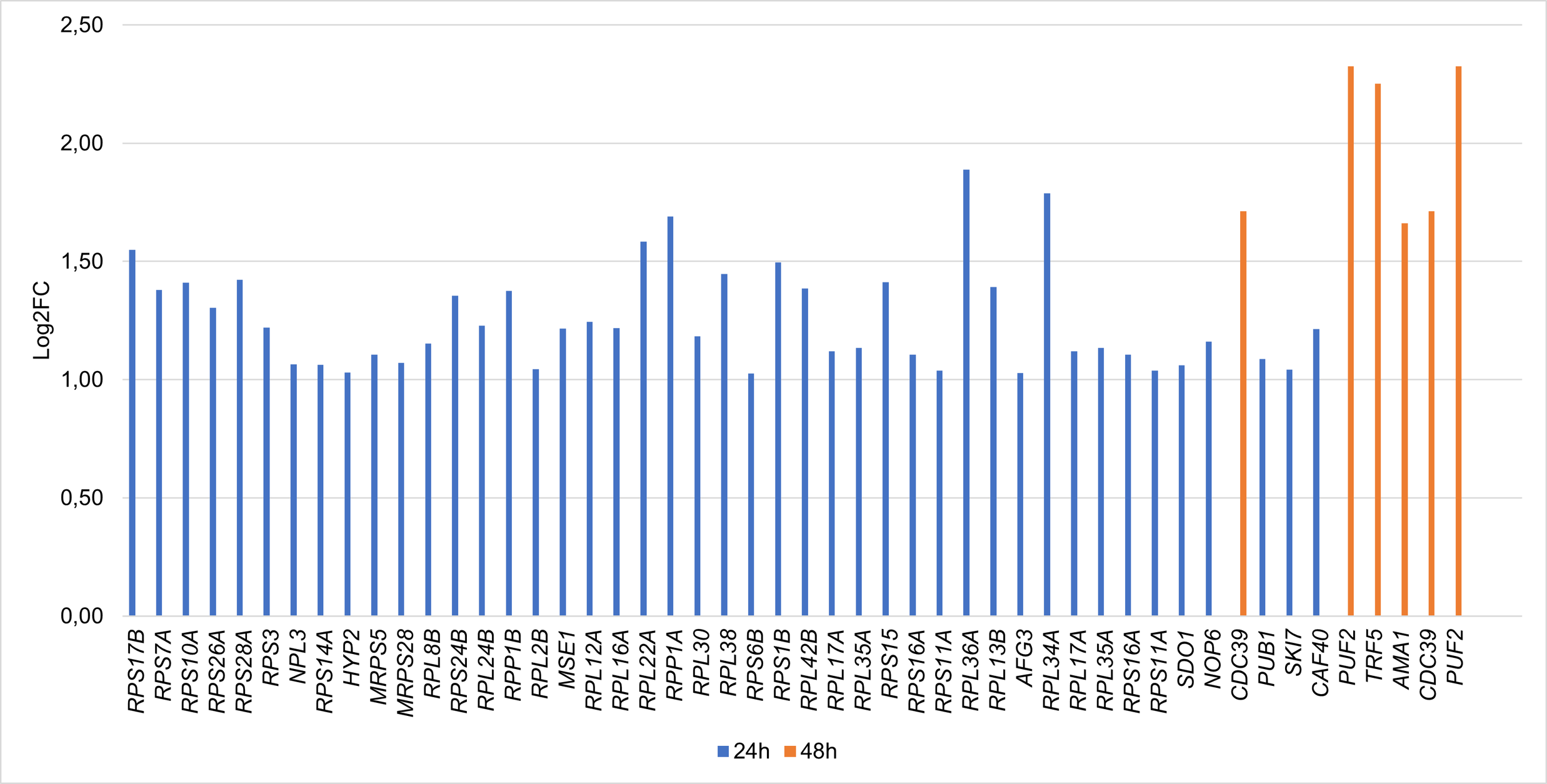

Supplement: S2 Fig — (TIF) [file pone.0316713.s002.tif]

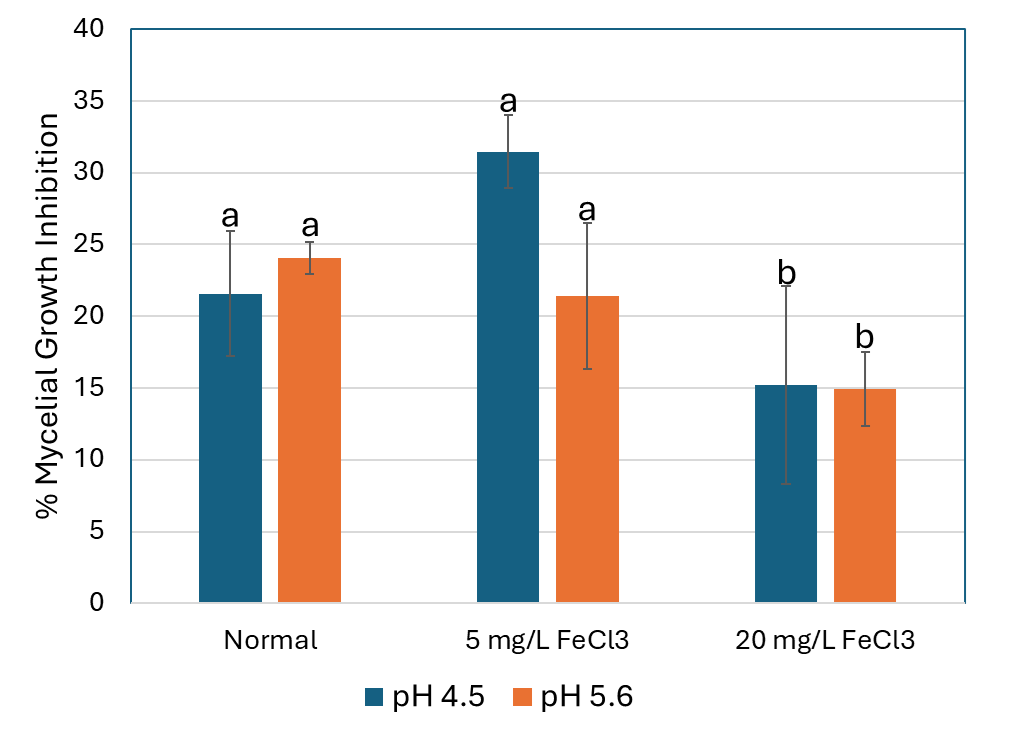

Supplement: S3 Fig — Different letters over the bars show significant differences between the iron concentrations according to Tukey’s post hoc test (p = 0.05). (TIF) [file pone.0316713.s003.tif]
