# Supplementary material for: Unravelling the transcriptomic dynamics of Hyphopichia pseudoburtonii in co-culture with Botrytis cinerea
Source: PLoS One. 2025 Jan 14;20(1):e0316713. doi: 10.1371/journal.pone.0316713 (PMC11731708; doi:10.1371/journal.pone.0316713)
Supplement: S1 Table — (DOCX) [file pone.0316713.s004.docx]

**S1 Table.** **Overview of the number of raw, quality trimmed/filtered and mapped reads for the interaction experiment of *H. pseudoburtonii* Y963 with *B. cinerea* FF1**

|  | **Treatment** | **No. of paired**  **raw reads** | **Trimmed and**  **quality filtered [%]** | **Total mapped to concatenated genome [%]** | **Mapped to**  ***H. pseudoburtonii* [%]** | **Mapped to**  ***B. cinerea* [%]** |
| --- | --- | --- | --- | --- | --- | --- |
| **24 h** | Monoculture | 18 382 957 | 97.1 | 79.04 | 75.63 | 0 |
|  |  | 19 296 254 | 97.6 | 70.55 | 68.41 | 0 |
|  |  | 20 712 865 | 97.7 | 51.14 | 55.53 | 0 |
|  | Co-cultures | 21 079 450 | 97.8 | 63.19 | 61.28 | 1.70 |
|  |  | 23 066 792 | 97.4 | 55.04 | 52.89 | 1.77 |
|  |  | 21 344 252 | 97.2 | 55.56 | 48.90 | 1.54 |
| **48 h** | Monoculture | 17 184 986 | 97.5 | 64.84 | 62.29% | 0 |
|  |  | 17 297 977 | 97.5 | 76.59 | 74.24% | 0 |
|  |  | 12 110 663 | 97.0 | 62.96 | 59.86 | 0 |
|  | Co-culture | 14 318 631 | 97.6 | 85.99 | 83.84 | 1.84 |
|  |  | 16 983 473 | 97.6 | 78.76 | 76.35 | 1.22 |
|  |  | 19 227 967 | 96.6 | 71.51 | 67.59 | 1.22 |
| **120 h** | Monoculture | 12 973 812 | 97.5 | 81.90 | 79.2 | 0 |
|  |  | 13 284 344 | 97.7 | 78.63 | 76.5 | 0 |
|  |  | 15 070 242 | 97.4 | 81.77 | 76.83 | 0 |
|  | Co-culture | 13 454 627 | 97.7 | 83.18 | 77.88 | 5.30 |
|  |  | 12 270 157 | 97.4 | 82.88 | 76.13 | 5.80 |
|  |  | 14 572 059 | 96.9 | 82.27 | 75.92 | 5.59 |
