# Supplementary material for: Unravelling the transcriptomic dynamics of Hyphopichia pseudoburtonii in co-culture with Botrytis cinerea
Source: PLoS One. 2025 Jan 14;20(1):e0316713. doi: 10.1371/journal.pone.0316713 (PMC11731708; doi:10.1371/journal.pone.0316713)
Supplement: S2 Table — (DOCX) [file pone.0316713.s005.docx]

**S2 Table. Log2 fold change of the top 15 downregulated genes in *H. pseudoburtonii* co-cultured with *B. cinerea* at 24, 48 and 120 h compared to the monoculture.**

| **Time** | **Gene name** | **Description/Function** | **Log2FC** |
| --- | --- | --- | --- |
| **24 h** | *Unknown* | Hypothetical protein | -2.36 |
|  | *Unknown* | Hypothetical protein | -2.18 |
|  | *Unknown* | Hypothetical protein | -2.15 |
|  | *FKS1* | FK506 Sensitivity | -2.02 |
|  | *ETP1* | Ethanol tolerance protein | -1.92 |
|  | *WHI4* | Putative RNA binding protein | -2.04 |
|  | *SPO14* | Phospholipase D | -1.80 |
|  | *Unknown* | Hypothetical protein | -1.80 |
|  | *RRP5* | Ribosomal RNA processing | -1.79 |
|  | *SSK2* | Suppressor of sensor kinase | -1.76 |
|  | *SOF1* | Suppressor of fibrillarin | -1.75 |
|  | *Unknown* | Hypothetical protein | -1.73 |
|  | *PDR5* | Pleiotropic drug resistance | -1.73 |
|  | *SLA1* | Cytoskeletal protein binding protein | -1.71 |
|  | *Unknown* | Hypothetical protein | -1.71 |
| **48 h** | *Unknown* | Hypothetical protein | -2.84 |
|  | *RAD50* | Radiation sensitive | -1.49 |
|  | *Unknown* | Hypothetical protein | -1.35 |
|  | *TEL1* | Telomere maintenance | -2.85 |
|  | *Unknown* | hypothetical protein | -1.89 |
|  | *THI13* | Thiamine metabolism | -1.52 |
|  | *SEO1* | Suppressor of sulfoxyde ethionine resistance | -1.21 |
|  | *Unknown* | hypothetical protein | -1.18 |
|  | *WSC3* | Cell wall integrity and stress response component | -2.29 |
|  | *URM1* | Ubiquitin related modifier | -1.61 |
| **120 h** | *Unknown* | Hypothetical protein | -1.28 |
|  | *DFG5* | Putative mannosidase | -1.13 |
|  | *SCW4* | Soluble cell wall protein | -1.20 |
|  | *PIN2* | Psi+ INducibility | -1.36 |
|  | *YCG1* | Subunit of the condensin comple | -1.17 |
|  | *SHS1* | Seventh homolog of septin | -1.53 |
|  | *Unknown* | hypothetical protein | -1.65 |
|  | *PMC1* | Vacuolar Ca2+ ATPase | -1.00 |
|  | *Unknown* | Hypothetical protein | -1.18 |
|  | *Unknown* | Hypothetical protein | -1.12 |
|  | *MRPL16* | Mitochondrial ribosomal protein of the large subunit | -1.16 |
|  | *OAC1* | Oxaloacetate carrier | -1.03 |
|  | *Unknown* | Hypothetical protein | -1.30 |
