# Supplementary material for: Unravelling the transcriptomic dynamics of Hyphopichia pseudoburtonii in co-culture with Botrytis cinerea
Source: PLoS One. 2025 Jan 14;20(1):e0316713. doi: 10.1371/journal.pone.0316713 (PMC11731708; doi:10.1371/journal.pone.0316713)
Supplement: S3 Table — (DOCX) [file pone.0316713.s006.docx]

**S3 Table.** **Significantly enriched KEGG pathways (q ≤ 0.05) of DEGs in *H. pseudoburtonii* grown for 24, 48 and 120 h in the presence of *B. cinerea* FF1**

| **Time** | **#term ID** | **term description** | **Gene no.** | **FDR** | **Gene list** |
| --- | --- | --- | --- | --- | --- |
| **24 h, up-regulated** | sce03010 | Ribosome | 27 | 4.19e-10 | *RPS6B, MRPS5,RPS14A,RPP1A,RPP1B,MRPS28,RPS17B,RPL34A,RPL30,RPS26A,RPL26B,RPL24B,RPL42B,RPL16A,RPS5,RPL17A,RPL8B,RPL22A,RPL38,RPS1B,RPL13B,RPL36A,RPS3,RPS15,RPS7A,RPS28A,RPS10A* |
|  | sce00190 | Oxidative phosphorylation | 10 | 0.0124 | *PMA1, COX13,VMA7,QCR9,COX6,ATP7,VMA5,ACP1,SDH2,VMA11* |
|  | sce04213 | Longevity regulating pathway – multiple species | 11 | 0.00015 | *GPR1, SIR2, GIS1,HSP78,RIM15,KOG1,SCH9,CYR1,TOR2,HSP104,MSN2* |
|  | sce04011 | MAPK signaling pathway – yeast | 14 | 0.0162 | *CYC8 ,STE5,HKR1,SLN1,BCK1,PBS2,SSK1,STT4,FKS1,MSN2,FKS3,YCK2,BNI1,SSK2* |
|  | sce02010 | ABC transporters | 5 | 0.0261 | *SNQ2, ,YCF1,YOR1,BPT1,PDR5* |
| **48 h, up-regulated** | sce04011 | MAPK signaling pathway – yeast | 5 | 0.0063 | *STE5,HKR1,SLN1,PBS2,SSK2* |
| **120 h, up-regulated** | sce01100 | Metabolic pathways | 44 | 2.03e-08 | *CDC19, ACS1, ATP1, ETR1, PHO5, IFA38, PGK1, THI13, CTA1, FAA2, ICL1, AGX1, POX1, THI4, ENO1, BIO2, TDH1, INO1, ACO2, CPA2, BAT2, MDH1, GPM1, FOX2, PDC5, CTS1, MET17, ACO1, CAR2, DAK1, ADH3, YMR226C, GAD1, MLS1, LYS9, BIO4, BIO3, ADH1, GCY1, ALD4, PMA2, THI6, TKL1, GPH1* |
|  | sce01110 | Biosynthesis of secondary metabolites | 28 | 3.35e-08 | *CDC19, ACS1, IFA38, PGK1, CTA1, ICL1, AGX1, POX1, ENO1, TDH1, INO1, ACO2, STE24, BAT2, MDH1, GPM1, PDC5, MET17, ACO1, CAR2, ADH3, GAD1, MLS1, LYS9, ADH1, ALD4, TKL1, GPH1* |
|  | sce01200 | Carbon metabolism | 17 | 3.35e-08 | *CDC19, ACS1, PGK1, CTA1, ICL1, AGX1, POX1, ENO1, TDH1, MDH1, GPM1, FOX2, MET17, ACO1, DAK1, MLS1, TKL1* |
|  | sce00010 | Glycolysis / Gluconeogenesis | 10 | 1.28e-05 | *CDC19, ACS1, PGK1, ENO1, TDH1, GPM1, PDC5, ADH3, ADH1, ALD4* |
|  | sce00630 | Glyoxylate and dicarboxylate metabolism | 7 | 0.00013 | *ACS1, CTA1, ICL1, AGX1, MDH1, ACO1, MLS1* |
|  | sce01230 | Biosynthesis of amino acids | 11 | 0.0014 | *CDC19, PGK1, ENO1, TDH1, ACO2, BAT2, GPM1, MET17, ACO1, LYS9, TKL1* |
|  | sce00071 | Fatty acid degradation | 5 | 0.0019 | *FAA2, POX1, ADH3, ADH1, ALD4* |
|  | sce00410 | beta-Alanine metabolism | 4 | 0.0040 | *POX1, FOX2, GAD1, ALD4* |
|  | sce00680 | Methane metabolism | 5 | 0.0040 | *ACS1, AGX1, ENO1, GPM1, DAK1* |
|  | sce04146 | Peroxisome | 6 | 0.0040 | *CTA1, FAA2, AGX1, POX1, SOD2, CAT2* |
|  | sce00780 | Biotin metabolism | 3 | 0.0062 | *BIO2, BIO4, BIO3* |
|  | sce00730 | Thiamine metabolism | 4 | 0.0096 | *PHO5, THI13, THI4, THI6* |
|  | sce01212 | Fatty acid metabolism | 4 | 0.0165 | *ETR1, IFA38, FAA2, POX1* |
|  | sce00620 | Pyruvate metabolism | 5 | 0.0228 | *CDC19, ACS1, MDH1, MLS1, ALD4* |
|  | sce00640 | Propanoate metabolism | 3 | 0.0328 | *ACS1, POX1, FOX2* |
