# Supplementary material for: Unravelling the transcriptomic dynamics of Hyphopichia pseudoburtonii in co-culture with Botrytis cinerea
Source: PLoS One. 2025 Jan 14;20(1):e0316713. doi: 10.1371/journal.pone.0316713 (PMC11731708; doi:10.1371/journal.pone.0316713)
Supplement: S4 Table — T-Test (p-value) and fold change (FC) were calculated in SCAFFOLD software. (DOCX) [file pone.0316713.s007.docx]

**S4 Table. Protein and clusters identified in *H. pseudoburtonii* monoculture and *H. pseudoburtonii* + *B. cinerea* co-cultures. T-Test (p-value) and fold change (FC) were calculated in SCAFFOLD software.**

| **Category** | **Identified Proteins** | **Accession Number** | **(*p*-value)** | **FC** |
| --- | --- | --- | --- | --- |
| **Translation and protein synthesis** | Cluster of 60S ribosomal protein L8 | A0A1E4RJ27_9ASCO [2] | 0.32 | 0.5 |
|  | 60S ribosomal protein L8 | A0A1E4RDS4_9ASCO | 0.23 | 0.5 |
|  | 60S ribosomal protein L8 | A0A1E4RJ27_9ASCO | 0.32 | 0.5 |
|  | Ribosomal protein S3 | A0A1E4RMQ2_9ASCO | 0.42 | 0.6 |
|  | 40S ribosomal protein S18 | A0A1E4REC5_9ASCO | 0.055 | 0.3 |
|  | 40S ribosomal protein S16* | A0A1E4RR23_9ASCO | 0.013 | 0.3 |
|  | 40S ribosomal protein S4 | A0A1E4RFM7_9ASCO (+1) | 0.27 | 0.4 |
|  | 60S acidic ribosomal protein P0 | A0A1E4RRL4_9ASCO | 0.41 | 0.6 |
|  | 60S ribosomal protein L27 | A0A1E4RRC6_9ASCO | 0.71 | 0.8 |
|  | Cluster of 40S ribosomal protein S1 | A0A1E4RQZ9_9ASCO [3] | 0.92 | 1 |
|  | 40S ribosomal protein S1 | A0A1E4RQZ9_9ASCO | 0.92 | 1 |
|  | 40S ribosomal protein S1** | A0A1E4RR15_9ASCO | 0.61 | 1.8 |
|  | 40S ribosomal protein S1 | RS3A_BOTFB | 0.22 | 0 |
|  | Ribosomal protein S4** | A0A1E4RPW6_9ASCO | 0.33 | 4.7 |
|  | 40S ribosomal protein S14 ** | A0A1E4RFC9_9ASCO | 0.41 | 1.6 |
|  | 40S ribosomal protein S6 | A0A1E4RMH4_9ASCO | 0.79 | 0.9 |
|  | 60S ribosomal protein L3 | A0A1E4RLB9_9ASCO | 0.89 | 1.1 |
|  | Elongation factor 2* | A0A1E4RI69_9ASCO | 0.025 | 0.3 |
|  | Translation elongation factor eEF-1 gamma chain | A0A1E4RT65_9ASCO | 0.14 | 0.4 |
|  | Ribosomal_S7 domain-containing protein | A0A1E4RDI3_9ASCO | 0.52 | 0.7 |
|  | Ribosomal protein | A0A1E4RTA8_9ASCO | 0.92 | 1 |
|  | Cluster of Translation elongation factor 1-alpha (Fragment) | E7CAA5_9ASCO [4] | 0.024 | 0.7 |
|  | Elongation factor 1-alpha (Fragment)* | A0A1B0Z732_9ASCO | 0.016 | 0.8 |
|  | Translation elongation factor 1-alpha (Fragment) | E7CAA5_9ASCO | 0.13 | 0.8 |
|  | Elongation factor 1-alpha (Fragment)* | A0A0D3RPB1_9ASCO | 0.049 | 0.8 |
|  | Elongation factor 1-alpha (Fragment) | A0A1B0Z725_9ASCO | 0.15 | 0.8 |
|  | EF-Tu/eEF-1alpha/eIF2-gamma C-terminal domain-containing protein (Fragment) | A0A1E4RFN8_9ASCO | 0.55 | 0.7 |
|  | Ribosomal protein L26A | A0A1E4RDS0_9ASCO | 0.27 | 0.8 |
| **Cell wall related** | Cell division control protein 48 | A0A1E4RS72_9ASCO | 0.16 | 0.1 |
|  | Cluster of Cell wall protein PhiA | A0A1E4RDM6_9ASCO [2] | 0.9 | 1 |
|  | Cell wall protein PhiA | A0A1E4RDM6_9ASCO | 085 | 0.9 |
|  | Cell wall-binding protein | A0A1E4RHG7_9ASCO | 0.94 | 1 |
|  | 1,3-beta-glucanosyltransferase | A0A1E4RJY8_9ASCO | 0.74 | 0.7 |
| **Glycolysis and Energy Metabolism:** | Cluster of Phosphopyruvate hydratase | A0A1E4RHG0_9ASCO [3] | 0.99 | 1 |
|  | Phosphopyruvate hydratase | A0A1E4RS55_9ASCO | 0.81 | 0.8 |
|  | Phosphopyruvate hydratase | A0A1E4RG59_9ASCO | 0.78 | 0.8 |
|  | Phosphopyruvate hydratase | A0A1E4RHG0_9ASCO | 0.99 | 1 |
|  | Alpha-amylase | A9ZPM0_9ASCO | 0.96 | 1 |
|  | Cluster of Glyceraldehyde-3-phosphate dehydrogenase | A0A1E4RGC3_9ASCO [3] | 0.89 | 1.1 |
|  | Glyceraldehyde-3-phosphate dehydrogenase | A0A1E4RKC1_9ASCO | 0.87 | 0.9 |
|  | Glyceraldehyde-3-phosphate dehydrogenase | A0A1E4RGC3_9ASCO | 0.91 | 1.1 |
|  | Glyceraldehyde-3-phosphate dehydrogenase | A0A1E4RGC3_9ASCO | 0.27 | 0 |
|  | Acetyl-coenzyme A synthetase | A0A1E4RK02_9ASCO | 0.94 | 1.1 |
|  | NAD-specific glutamate dehydrogenase | A0A1E4REM8_9ASCO | 0.24 | 0.2 |
|  | Fructose-bisphosphate aldolase | A0A1E4RGA3_9ASCO | 0.18 | 0.5 |
|  | Glycoside hydrolase* | A0A1E4RPI3_9ASCO | 0.011 | 0.5 |
|  | Biotin carboxylase | A0A1E4RLA8_9ASCO | 0.45 | 0.5 |
|  | H(+)-transporting two-sector ATPase | A0A1E4RQA6_9ASCO | 0.33 | 0.1 |
|  | Fatty acid synthase subunit beta | A0A1E4RJ85_9ASCO | 0.39 | 0.4 |
|  | ATP synthase subunit beta | A0A1E4RQM9_9ASCO | 0.52 | 0.6 |
|  | Adenosylhomocysteinase | A0A1E4RJG7_9ASCO | 0.47 | 0.6 |
|  | 5-methyltetrahydropteroyltriglutamate--homocysteine S-methyltransferase | A0A1E4RFE0_9ASCO | 0.6 | 0.6 |
|  | Phosphoglucomutase (alpha-D-glucose-1,6-bisphosphate-dependent)* | A0A1E4RCR6_9ASCO | 0.049 | 0.7 |
|  | Phosphoglycerate kinase | A0A1E4RQZ5_9ASCO | 0.51 | 0.7 |
|  | Phosphomannomutase | A0A1E4RS74_9ASCO | 0.67 | 0.8 |
|  | Fatty acid synthase subunit alpha | A0A1E4RNE5_9ASCO | 0.54 | 0.8 |
|  | 6-phosphogluconate dehydrogenase, decarboxylating | A0A1E4RKB6_9ASCO | 0.83 | 0.9 |
|  | Triosephosphate isomerase | A0A1E4RQZ7_9ASCO | 0.72 | 0.8 |
|  | Pyruvate decarboxylase | A0A1E4RBR2_9ASCO | 0.68 | 0.8 |
|  | ADH1 | sp\|ADH1_YEAST\| | 0.86 | 0.9 |
|  | Xylitol dehydrogenase | A0A1E4RGQ2_9ASCO | 0.84 | 0.9 |
|  | Acetyl-coenzyme A synthetase | A0A1E4RK02_9ASCO | 0.94 | 1.1 |
|  | Cluster of Exocyst complex component SEC10 | A0A1E4RKQ0_9ASCO [3] | 0.91 | 1.1 |
|  | Exocyst complex component SEC10 | A0A1E4RKQ0_9ASCO | 0.91 | 1.1 |
|  | D-xylose reductase (NAD(P)H)** | A0A1E4RLY1_9ASCO | 0.75 | 1.2 |
|  | Phosphoglycerate mutase ** | A0A1E4RM70_9ASCO | 0.59 | 1.2 |
|  | Six-hairpin glycosidase (Fragment)** | A0A1E4RGA6_9ASCO | 0.81 | 1.2 |
|  | DUF3757 domain-containing protein** | A0A1E4REI4_9ASCO | 0.62 | 1.3 |
|  | Plasma membrane ATPase* | A0A1E4RI84_9ASCO | 0.66 | 1.5 |
|  | Chaperonin GroL ** | A0A1E4RHK0_9ASCO | 0.69 | 1.6 |
|  | Transaldolase ** | A0A1E4REE9_9ASCO | 0.61 | 1.6 |
|  | Alpha-1,4 glucan phosphorylase ** | A0A1E4RED5_9ASCO | 0.4 | 2.2 |
|  | Pyruvate kinase ** | A0A1E4RTD9_9ASCO | 0.25 | 2.3 |
|  | ATP synthase subunit alpha ** | A0A1E4RJW3_9ASCO | 0.35 | 2.3 |
|  | Formate dehydrogenase | A0A1E4RPW7_9ASCO | 0.27 | 0 |
| **Heat shock proteins and stress response** | HSP90-domain-containing protein | A0A1E4RQA4_9ASCO | 0.76 | 0.8 |
|  | Heat shock protein 70 | A0A1E4RCG8_9ASCO | 0.51 | 0.7 |
|  | Heat shock protein 70, Hsp70 family | A0A1E4RK48_9ASCO | 0.9 | 1.1 |
|  | Heat shock protein 70 | A0A1E4RRH9_9ASCO | 0.9 | 1.1 |
|  | Heat shock protein 70* | A0A1E4RQG3_9ASCO | 0.028 | 0.2 |
|  | GroES-like protein | A0A1E4RKX5_9ASCO | 0.72 | 0.9 |
|  | GroES-like protein | A0A1E4RFJ5_9ASCO | 0.57 | 0.6 |
|  | Peroxiredoxin TSA1 | A0A1E4RKL6_9ASCO | 0.91 | 0.9 |
|  | Mitochondrial heat shock protein of the HSP70 family | A0A1E4RNX5_9ASCO | 0.86 | 0.8 |
| **Signaling and regulation** | ARM repeat-containing protein | A0A1E4RNT0_9ASCO | 0.46 | 0.6 |
| **Miscellaneous** | Protein TOS1 ** | A0A1E4RQ57_9ASCO | 0.059 | 3.3 |
|  | PR-1-like protein** | A0A1E4RCA1_9ASCO | 0.5 | 1.2 |
|  | Flavo protein WrbA | A0A1E4RQV6_9ASCO | 0.92 | 1 |

The proteins marked with an asterisk (*) are those uniquely found in the co-culture (Hp+B), and those marked with a double asterisk (**) are those with a fold change ≥1.2, indicating they are differentially abundant proteins (DAPs). These DAPs show significant changes in abundance in response to the presence of *B. cinerea*.
